# *OsLG3* contributing to rice grain length and yield was mined by Ho-LAMap

**DOI:** 10.1186/s12915-017-0365-7

**Published:** 2017-04-06

**Authors:** Jianping Yu, Haiyan Xiong, Xiaoyang Zhu, Hongliang Zhang, Huihui Li, Jinli Miao, Wensheng Wang, Zuoshun Tang, Zhanying Zhang, Guoxin Yao, Qiang Zhang, Yinghua Pan, Xin Wang, M. A. R. Rashid, Jinjie Li, Yongming Gao, Zhikang Li, Weicai Yang, Xiangdong Fu, Zichao Li

**Affiliations:** 1 Key Laboratory of Crop Heterosis and Utilization, Ministry of Education/Beijing Key Laboratory of Crop Genetic Improvement, China Agricultural University, Beijing, 100193 China; 2 Institute of Crop Sciences, Chinese Academy of Agricultural Sciences, Beijing, China; 3 Institute of Genetics and Developmental Biology, Chinese Academy of Sciences, Beijing, China; 4 Rice Research Institute, Guangxi Academy of Agricultural Sciences, Nanning, Guangxi China

**Keywords:** GWAS, Linkage mapping, Observed heterozygosity, Ho-LAMap, *OsLG3*, Grain length, Rice domestication, Artificial selection, Genetic interaction

## Abstract

**Background:**

Most agronomic traits in rice are complex and polygenic. The identification of quantitative trait loci (QTL) for grain length is an important objective of rice genetic research and breeding programs.

**Results:**

Herein, we identified 99 QTL for grain length by GWAS based on approximately 10 million single nucleotide polymorphisms from 504 cultivated rice accessions (*Oryza sativa* L.), 13 of which were validated by four linkage populations and 92 were new loci for grain length. We scanned the Ho (observed heterozygosity per locus) index of coupled-parents of crosses mapping the same QTL, based on linkage and association mapping, and identified two new genes for grain length. We named this approach as Ho-LAMap. A simulation study of six known genes showed that Ho-LAMap could mine genes rapidly across a wide range of experimental variables using deep-sequencing data. We used Ho-LAMap to clone a new gene, *OsLG3*, as a positive regulator of grain length, which could improve rice yield without influencing grain quality. Sequencing of the promoter region in 283 rice accessions from a wide geographic range identified four haplotypes that seem to be associated with grain length. Further analysis showed that *OsLG3* alleles in the *indica* and *japonica* evolved independently from distinct ancestors and low nucleotide diversity of *OsLG3* in *indica* indicated artificial selection. Phylogenetic analysis showed that *OsLG3* might have much potential value for improvement of grain length in *japonica* breeding.

**Conclusions:**

The results demonstrated that Ho-LAMap is a potential approach for gene discovery and *OsLG3* is a promising gene to be utilized in genomic assisted breeding for rice cultivar improvement.

**Electronic supplementary material:**

The online version of this article (doi:10.1186/s12915-017-0365-7) contains supplementary material, which is available to authorized users.

## Background

Rice (*O. sativa* L.) is a staple food and the world’s most important cereal crop. Grain yield is determined by three component traits, namely grain weight, number of grains per panicle, and number of panicles per plant. Grain size is a prime breeding target, as it affects both yield and quality. Therefore, the study of grain size is highly important for the improvement of rice yield and quality as well as for the understanding of the rice domestication process [1]. Grain size is specified by its three dimensions (length, width, and thickness). Recently, although a number of quantitative trait loci (QTL) conferring grain length [2] have been isolated, and among them several QTL, such as *GS3*, *GW2*, *GL3.1*, *TGW6*, *qSW5*, and *GW8*, have been well studied [1, 3–7], molecular characterization of these and many more unknown genes is still largely unclear. Thus, it is of great significance to understand the underlying genetic and molecular bases of grain length [3].

Recent studies isolated and characterized genes involved in QTL using map-based cloning techniques based on linkage mapping [2, 6, 8–11]. However, for fine mapping, very large sample sizes are required; typically, thousands of individuals are needed and the fieldwork involved is extremely laborious, usually involving measurement of multiple traits at several time points across diverse environments. Genome-wide association analysis (GWAS) is generally considered an effective tool to infer causative links between genomic markers and phenotype in many crops [12–16]. However, in previous studies, the causal polymorphisms showed a weaker association than the peak single nucleotide polymorphism (SNP) in *Arabidopsis thaliana* and rice [12, 17]. Extensive computer simulations have shown that the power of GWAS is low for polygenic traits and that spurious associations can be expected [18]. Linkage mapping was shown to be a valuable complementary approach to address these situations in maize and Arabidopsis [14, 16, 19]. Recently, there have been attempts to combine the vigor of linkage mapping with GWAS in rice [20–22]. GWAS should be performed in conjunction with genetic linkage analysis to detect relevant loci [17].

It is generally agreed that GWAS in rice cannot resolve a single gene due to the low rate of linkage disequilibrium decay. With the development of next generation sequencing technology, DNA sequencing has become easier and cheaper. A core collection of 3000 rice accessions from 89 countries were deep resequenced [23, 24], providing an unprecedented resource for genomic research. Herein, we propose a unified approach for gene discovery in rice from candidate region association mapping combing linkage mapping that limits the major problem caused by false positives. We not only describe a new strategy to identify previously unknown but agronomically important alleles based on deep-sequencing technology, but also report on the cloning and characterization of a dominant QTL, *OsLG3*, as a positive regulator of grain length. Natural variations in the *OsLG3* promoter region confer grain length and weight and its favorable allele represents a valuable genetic resource for rice cultivar improvement.

## Results

### Ninety-nine QTL for grain length were detected by GWAS based on high-density SNPs

**Fig. 1:**
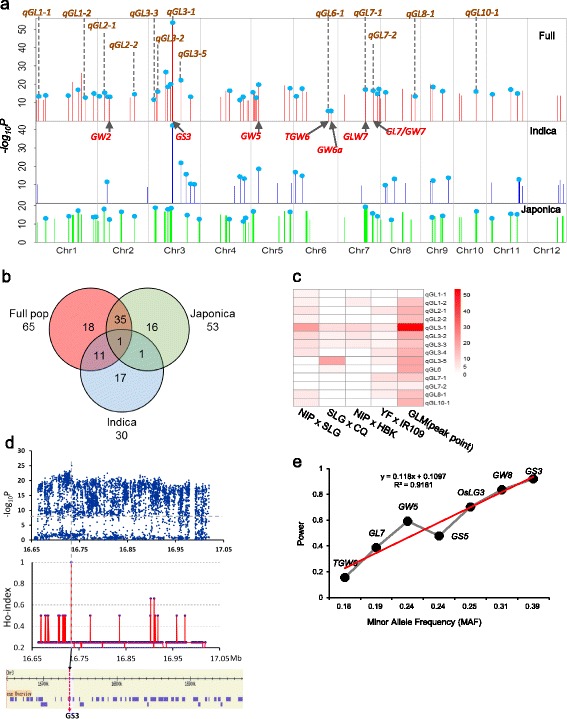
Ho-LAMap accelerated the identification of grain size loci in rice. **a** Comparison of GWAS results for grain length among the full, *indica*, and *japonica* populations using the GLM (Q) model. *Arrows* indicate the physical positions of known grain length genes overlapping with the QTL. Vertical dashed lines represent QTL co-localized with linkage mapping. *Blue dots* indicate QTL overlapping with the published QTL. **b** Venn diagram showing numbers of unique and shared QTL mapped by GWAS among the full, *indica*, and *japonica* populations. **c** Heat map for effect of grain length QTL mapped by four linkage populations and GWAS. Rows in the heat map correspond to the 14 QTL for grain length. **d** Identification of causal SNPs in QTL *qGL3-1* using Ho-LAMap, a novel method, which combined linkage mapping and association mapping for grain length. Manhattan plot for candidate region association mapping for *qGL3-1* (*top*) and Ho index plots of *qGL3-1* (*middle*). Red regression lines were obtained by averaging SNP indices from a moving window of two consecutive SNPs, and shifting the window one SNP at a time. The bottom part corresponds to annotated genes in this region, with the *purple arrow* representing *GS3*. **e** Minor allele frequency influence on effectiveness of Ho-LAMap

We investigated grain length in a diverse panel of 504 cultivated rice varieties from worldwide sources (Additional file 1: Figure S1 and Additional file 32: Table S1, Additional file 33: Table S2) grown in five environments at two different latitudes (Additional file 33: Table S3). The panel consisted of the mini core collection [25] and varieties in the International Rice Molecular Breeding Network [26] and have, as part of the rice 3000 genome project, been sequenced with an average sequencing depth of 14.9× (Data access). After aligning the reads against the rice reference genome of the temperate *japonica* variety, Nipponbare, we identified a total of 10 million SNPs (1 SNP per 40 bp on average) from these accessions and 3,585,229 SNPs with missing rates of less than 30% and a 0.05 minor allele frequency (MAF). GWAS was performed on different populations using the naïve model (LM), general linear model (GLM) with population structure (Q matrix), and compressed mixed linear model (CMLM) by controlling population structure and kinship, denoted by the Q + K model according to Yu et al. [27, 28] (Additional file 3: Figure S3a–c, Additional file 34: Table S5, Additional file 35: Table S6, Additional file 36: Table S7, Additional file 37: Table S8, and Methods). LM not only detected all the cloned genes and published QTL related to rice grain length, but also indicated a whole genome association signal in all types of populations, and thus included a very high number of false positive associations (Additional file 3: Figure S3a). When using CMLM and setting –log_10_ (*P*) = 7.9 (*P* = 0.05/n, n denotes the number of all SNPs), we detected only two, two, and four QTL in the full, *indica*, and *japonica* populations, respectively, of which one is the cloned gene *GS3*, one is a published QTL in both full and *indica* populations, and one is a published QTL in *japonica* (Additional file 3: Figure S3b and Additional file 34: Table S5). When using GLM and the same –log_10_ (*P*), we detected 65, 30, and 53 QTL in the full, *indica*, and *japonica* populations, respectively, and these QTL hit seven (*GW2*, *GS3*, *GW5*, *GW6a*, *TGW6*, *GL7*, and *GLW7*), two (*GS3* and *GW5*), and three (*GW2*, *GL7*, and *GLW7*) cloned genes, respectively, and about 65%, 53%, and 57% of the published QTL (Fig. 1a, b and Additional file 35: Table S6, Additional file 36: Table S7, Additional file 37: Table S8). However, four genes from map-based cloning (*GS2*, *GL3.1*, *GS5*, and *GW8*) were not identified by the model because of the rarity of causal variants, or strong correlation between causal variation and population structure. Most of the genes being separated by map-based cloning involve rare causal variations and cannot be identified by GWAS, such as *GS2* and *GL3.1* (Additional file 33: Table S9). Some other genes cannot be detected by GLM because the frequency of their causal variation confound with population structure (Additional file 4: Figure S4). For example, even though *GW8* and *GS5* displayed the high association signals in LM (–log_10_ (*P*) = 19.0 and 17.8), their signal in GLM decreased to 1.2 and 4.9, and *GW8* indicated no significance by simple t-test when removing effect of Q structure from their original phenotypes (Additional file 5: Figure S5). Taken together, these results suggested that there were still many valuable genes for grain length that remained to be cloned.

### Co-localized QTL for grain length by joining GWAS and multiple biparental linkage analysis

We used four segregating populations from cross involving six varieties to identify QTL for grain size and grain weight, including SLG-1 (SLG, with the largest grain), Chuanqi (CQ, with the smallest grain), Nipponbare (NIP, medium grain), Haobuka (HBK, large grain), IRAT109 (large grain), and Yuefu (YF, medium grain), which were selected from the MCC1 panel and showed high genetic differentiation from each other (Additional file 7: Figure S7 and Additional file 33: Table S10). The four segregating populations included BILs (backcross inbred lines) from Nipponbare and SLG-1, CSSLs (chromosome segregation substitution lines) from YF and IRAT109, BC_1_F_2_ lines from Chuanqi and SLG-1, and BC_1_F_2_ lines from Nipponbare and Haobuka. Substantial variation in grain size traits was observed among these populations (for example, 07DH010-14, Additional file 8: Figure S8). Thirty-four QTL for grain shape and weight were detected among the four groups (Additional file 9: Figure S9 and Additional file 33: Table S11). Among them, 13 QTL for grain length co-localized with QTL mapped by our GWAS (Fig. 1a, c). Of 34 QTL, 22 were detected in at least two combinations. Moreover, three QTL (*qGL3-1*, *qGL3-2*, and *qGL3-3*) for grain length were detected in four crosses (Fig. 1c).

### The efficiency of Ho-LAMap for detection of causal genes and simulation studies

Similar to other reports of GWAS in rice [12, 13, 29], the peak SNPs were not part of the causal genes except for *GS3*, even though we narrowed the QTL regions of several hundred kilobases (Additional file 6: Figure S6). However, analysis of the causal alleles of some cloned genes indicated that most of the varieties shared the same causal variations [5–7], although the varieties did not uniformly share the same non-causal variations. Based on this, we propose a strategy – Ho-LAMap that joins GWAS and multiple biparental linkage analysis – to distinguish the causal gene from other genes confounded with population structure. The principles of Ho-LAMap are depicted in Additional file 12: Figure S12 and Additional file 33: Notes S3. Firstly, we cross diverse founder varieties (i.e., varieties that are significantly different from reference parent for grain traits) with a reference parent (usually a small grain variety). After backcrossing or several self-pollinated generations, advanced populations are prepared for QTL mapping and these serve to identify the regions in the genome that are most likely to carry the causal genes. Primary fine mapping will help to ensure the boundary of the QTL. Association mapping using the GLM (Q) model in the QTL interval will identify almost all SNPs that correlate significantly with the target trait. In crosses that have detected the targeted QTL, the majority of SNPs within the QTL interval will segregate in a 1:1 founder varieties:reference parent ratio. However, the SNP responsible for the change of phenotype will be the same in all founder parents detecting the targeted QTL. If we define the Ho (observed heterozygosity per locus) index as the ratio between the number of heterozygous crosses corresponding to each SNP locus and the total number of crosses which have the targeted QTL, we expect this index to equal 1 near the causal SNP and 0.5 for the unlinked loci. Ho indices can be scanned across the genome to find the region with a Ho index of 1, and presumably harboring the gene responsible for the change of phenotype. In this method, we just need to sequence several parents and do SNP mapping. Our proposed name for this approach is Ho index unified linkage and association mapping (Ho-LAMap) as applied to rice.

Among three co-localized QTL, *qGL3-1* encompassed the cloned gene *GS3* [3] and thus was used to validate the efficiency of Ho-LAMap. In the mapping region of *qGL3-1* overlapping between GWAS and multiple biparental linkage analysis, we detected 1763 SNPs with significant association signals. For each identified locus, we obtained the Ho index between each pair of parents for the four combinations, and plotted the Ho indices in the overlapping mapped region (Fig. 1d). The Ho index of *GS3* was 1 and those for almost all other loci were below 0.5, and even 0. The results confirmed that this approach allowed us to rapidly identify the causal gene according to GWAS combined with primarily mapped QTL common among multiple biparental crosses.

Given the success Ho-LAMap in identifying genes controlling quantitative traits based on common QTL and GWAS, we are more generally interested in the possible factors affecting the efficiency of Ho-LAMap in detecting causal genes. Simulation studies (see Methods) on several known genes, such as *GS3*, *TGW6*, etc., were carried out to estimate (1) the number of crosses required (N), (2) the minor allele frequency of the targeted gene, and (3) the subgroup that selected parents were from. Our results suggest that the minor allele frequency is perhaps the most important factor for effectiveness of Ho-LAMap and that parents selected from an appropriate subgroup would increase the efficiency (Fig. 1e and Additional file 13: Figure S13). Whereas the resolving power increased with the number of crosses we concluded that, in application of Ho-LAMap to complex quantitative traits in rice, N ≥ 4 should be required for genes such as *TGW6* (Additional file 14: Figure S14 and Additional file 33: Table S12).

### Identification of two new grain length loci via Ho-LAMap

**Fig. 2:**
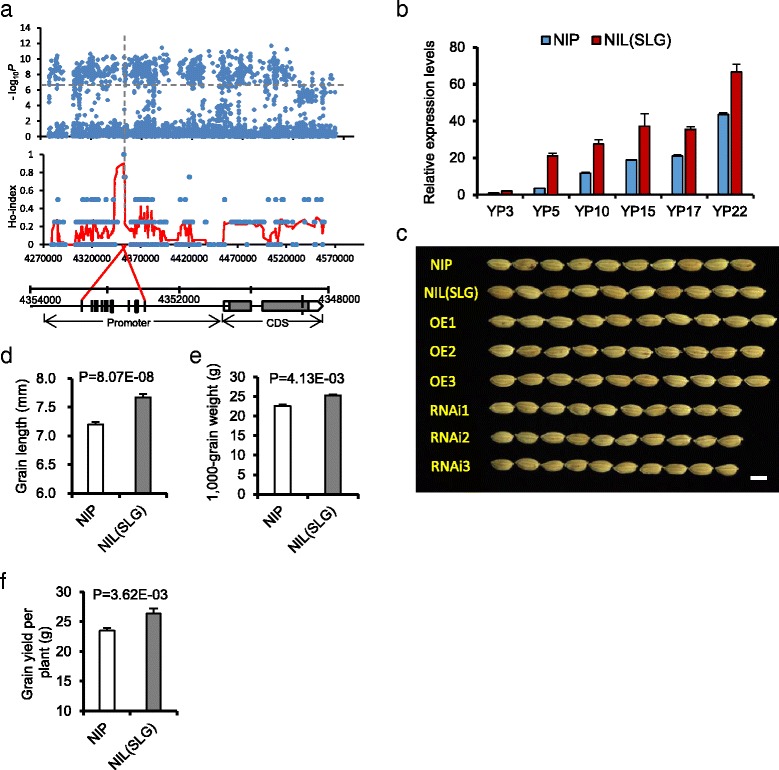
*OsLG3* mined by Ho-LAMap and its functional validation. **a** Identification of the causal SNPs of QTL *qGL3-3* using Ho-LAMap. Manhattan plot for candidate region association mapping for *qGL3-3* (*top*) and Ho index plots for *qGL3-3* (*middle*). The bottom region corresponds to the candidate gene (*OsLG3*), the red lines label the region for significant signal. **b** *OsLG3* expression levels in developing panicles from NIP and NIL(SLG) plants. YP3-YP22, young panicles at 3–22 cm in length; *n* = 3. **c** Grains from NIP, NIL(SLG), three *OsLG3*-overexpressing lines and three RNAi-*OsLG3* lines. Scale bar, 5 mm. Grain length (**d**), 1000-grain weight (**e**), grain yield per plant (**f**) of NIP and NIL(SLG) plants. All phenotypic data in **d–f** were measured from plants grown with 20 × 20 cm spacing in paddies under normal cultivation conditions. Data represent mean ± SEM (*n* = 30). Student’s *t* tests were used to generate *P* values

We further applied Ho-LAMap to *qGL3-2* and *qGL3-3* and successfully identified two new genes for grain length (Fig. 2a, Additional file 15: Figure S15 and Additional file 33: Notes S1, S2). Herein, we focused on *qGL3-3* and identified a small genomic region harboring a cluster of SNPs with a Ho index of 1 (Fig. 2a): *qGL3-3* showed a cluster of five SNPs with a Ho index of 1. Then, we examined the SNPs with Ho indices of 1 in detail. All five SNPs were localized to the promoter of gene *Os03g0183000* (*OsLG3*) (Additional file 33: Table S13), which encodes an APETALA2/ethylene-responsive element binding protein 125. We compared genomic sequences corresponding to the ORF and the promoter regions of *OsLG3* between varieties with short and long grain and found that the promoter region had high nucleotide diversity (Additional file 16: Figure S16). Significantly, three varieties with long grains (IRAT109, SLG-1, and Haobuka) had the same promoter sequences that were different from those of all short-grain varieties (Additional file 33: Table S14). We assayed the temporal and spatial expression patterns of *OsLG3* in NIL(SLG) and NIL(NIP) using quantitative real-time PCR (qRT-PCR) with total RNA from 13 tissues. The transcript was much more abundant in NIL(SLG) than in NIL(NIP) in young panicles and developing rice endosperms (Fig. 2b and Additional file 17: Figure S17). Such expression differences corresponded well with the critical stages for determination of grain length and grain weight [30]. Therefore, we hypothesized that differences in expression levels of *OsLG3*, attributable to polymorphisms in the promoter rather than the coding variation, were key determinants of variation in grain length.

Two transformation constructs were prepared to test this hypothesis. The first, OE, contained the cDNA of *OsLG3* from Nipponbare (short grain) driven by the 35S promoter; the second, contained RNAi-*OsLG3*, the fragment of *OsLG3* coming from Nipponbare. All of the transgenic lines that overexpressed the Nipponbare *OsLG3* allele showed increased grain length and grain weight, compared to transgene-negative plants (Fig. 2c and Additional file 18: Figure S18a–c). All transgenic RNAi-*OsLG3* plants formed grains that were substantially shorter and lighter than those from transgene-negative plants (Fig. 2c and Additional file 18: Figure S18d, e). Thus, a longer grain could be caused by the variations in the promoter region.

### *OsLG3* regulates grain length by altering cell number but does not influence grain quality

Although most of the morphological characteristics of NIL(SLG) were similar to those of Nipponbare (Additional file 19: Figure S19), the spikelet hulls of NIL(SLG) plants before fertilization were longer than those of Nipponbare plants. We conducted scanning electron microscopy study of outer and inner surfaces of glumes from NIL(SLG) and NIP (Additional file 20: Figure S20). There was little, if any, difference in cell length in either the palea or lemma, but total cell numbers of outer and inner epidermal cells in the longitudinal direction in NIL(SLG) were more than their equivalents in Nipponbare. We also investigated the expression of the key genes determining cell cycle time [7, 31] such as *CDKA1*, *CYCD3*, *MCM3*, *CYCA2.1*, *CYCA2.2*, *CYCA2.3*, *CAK1*, and *H1*. The transcript levels of most of these genes were considerably higher in NIL(SLG) plants relative to Nipponbare plants (Additional file 21: Figure S21), suggesting that the increase in cell number in NIL(SLG) might result from elevated expression of genes promoting cell proliferation. In addition, compared with Nipponbare, NIL(SLG) had a 4.2% higher grain length to width ratio, but had the same grain chalkiness level and the same starch granule appearance in transverse sections of grains (Additional file 22: Figure S22). These observations suggest that *OsLG3* might promote longitudinal growth by increasing cell proliferation while not influencing grain quality (Fig. 2d–f and Additional file 23: Figure S23 and Additional file 24: Figure S24).

### Expression patterns of *OsLG3* and its transcription activator activity

As mentioned above, *OsLG3* transcripts were detected in various tissues by qRT-PCR analysis (Fig. 2b and Additional file 17: Figure S17). To determine the spatial expression pattern of *OsLG3* in detail, we generated transgenic plants containing *OsLG3* promoter::GUS fusions (*ProOsLG3::GUS*). We observed GUS activity in all of the analyzed tissues/organs (roots, stems, sheaths, leaves and panicles) (Additional file 25: Figure S25). Higher GUS activity was detected in developing panicles, spikelet hulls during spikelet development and roots (Additional file 25: Figure S25). These results indicate that *OsLG3* is a temporally and spatially expressed gene.

**Fig. 3:**
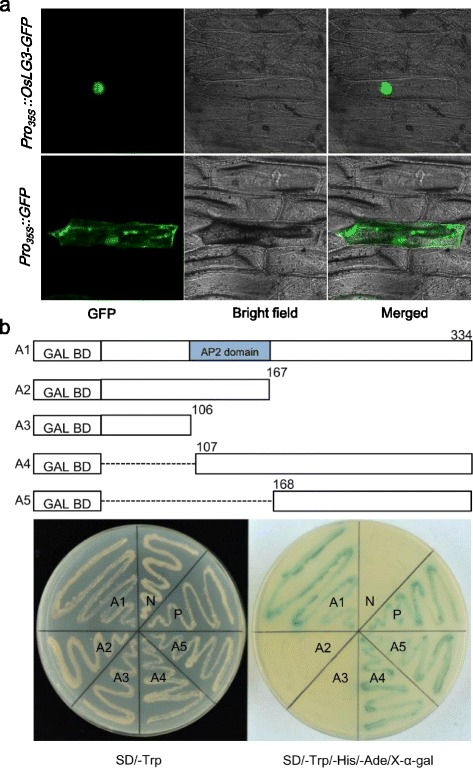
*OsLG3* is a nuclear-localized transcriptional activator. **a** Subcellular localization of *OsLG3*. **b** Transactivation assay of truncated *OsLG3*. Fusion proteins of the GAL4 DNA-binding domain and different portions of *OsLG3* were expressed in yeast strain AH109. A1 indicates the full-length coding sequence of *OsLG3*; A2 to A5 indicate the mutated forms of *OsLG3* (nucleotide positions were labeled in the diagrams), respectively. P and N indicate the positive and negative control, respectively. The culture solution of the transformed yeast was dropped onto the control plate SD/-Trp or selective plate (SD/-Trp/-Ade/-His/X-α-gal). The plates were incubated for 3 days

As *OsLG3* encodes an AP2 domain class transcription factor, we speculated that *OsLG3* is localized in the nucleus. To determine subcellular localization of *OsLG3*, we expressed an *OsLG3*-green fluorescent protein (GFP) fusion protein under the control of the 35S promoter in onion epidermal cells. As shown in Fig. 3a, GFP fluorescence in 35S: *OsLG3*-GFP transgenic epidermal cells was observed exclusively in nuclei. Thus, this result suggests that *OsLG3* is a nuclear-localized protein, consistent with its proposed function as a transcription regulator.

We further tested the activity of a set of truncations and deletions of *OsLG3/OsAP2-125*. It showed that the C-terminal 168–334 aa region is sufficient to activate the reporter, whereas the N-terminal truncated *OsLG3/OsAP2-125* proteins are not (Fig. 3b). These results show that transcription activation activity of *OsLG3/OsAP2-125* resides in its C-terminal regions and that DNA-binding activity resides in its N-terminal regions containing the AP2 domain [32, 33].

### Validation of functional variations in promoter region and origin of *OsLG3*

**Fig. 4:**
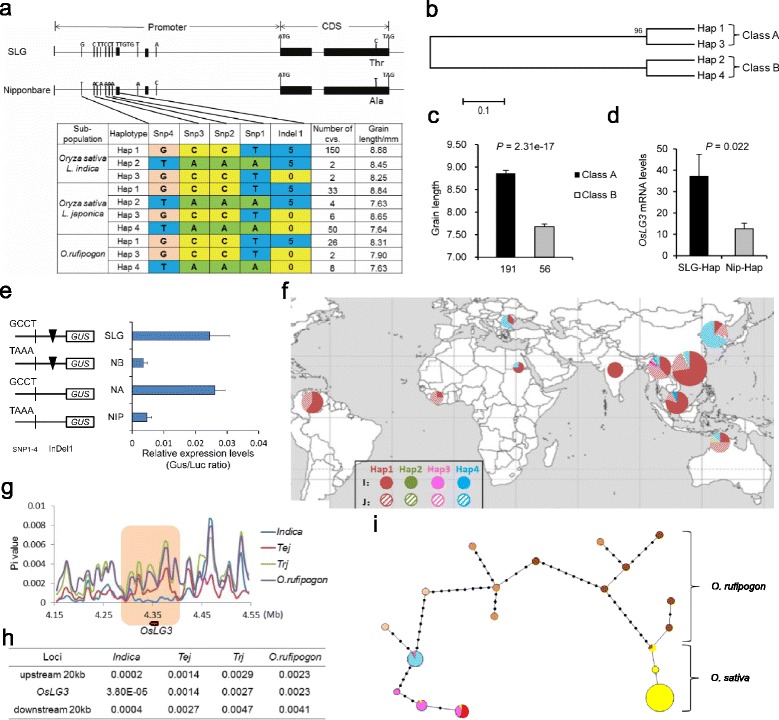
Functional variation, the haplotypes and origin of *OsLG3*. **a** Haplotype analysis of *OsLG3*. Gene structure and natural variation between alleles from SLG and Nipponbare (*top*). Natural variation in *OsLG3* among 283 rice accessions of a mini-core collection compared with the NILs (*bottom*). **b** Cladogram of four haplotypes. **c** Grain lengths of accessions for the two classes in MCC1; raw data are provided in Additional file 32: Table S1; n, is the number of accessions. *P* values were generated by two-tailed *t* tests. Error bars, SEM. **d** Relative expressions of *OsLG3* in two haplotypes (SLG-Hap, n = 17; Nip-Hap, n = 16). **e** Transient expression assays of the effect of the four consensus SNPs and one indel on gene expression. Relative firefly luciferase activities in Arabidopsis protoplasts, with data normalized to activity of co-transformed constitutively expressed *Renilla* luciferase. Data are shown as means ± SEM (n = 5 technical replicates). **f** Geographic origin of rice containing the SLG-type *OsLG3*. Hap1–4 are represented by red, green, purple, and blue circles, respectively. The *indica* and *japonica* cultivars are denoted by solid and dashed circles, respectively. **g** Nucleotide diversity analysis in *OsLG3* and flanking regions (~400 kb). Representative samples included 99 *indica* accessions, 68 *temperate japonica* accessions, 27 *tropical japonica* accessions, and 10 wild rice accessions (Additional file 32: Table S1). Tej, *temperate japonica*; trj, *tropical japonica*. Y-axis, average π value; x-axis, Nipponbare TIGR v7.0 genome coordinate of chromosome 3. **h** Averaged nucleotide diversity of the 20 kb surrounding *OsLG3*. **i** A minimum-spanning tree for the *OsLG3* region. Each haplotype group is represented by a circle, and circle size represents the number of lines within the haplotype, as in Fig. 4 f. Orange, brown, yellow, red, blue and pink represent *O. rufipogon* in China, *O. rufipogon* in Southeast Asia, *indica*, *tropical japonica*, *temperate japonica*, and *template japonica* with glutinous varieties from the Yunnan-Guizhou Plateau or mixtures with recently improved *indica* or *tropical japonica*. Grid corresponds to *oslg3/oslg3*

We next sequenced the *OsLG3* promoter regions and measured grain length in 283 rice accessions, including 247 cultivated, worldwide varieties of *O. sativa* in MCC1, and 36 wild accessions originating from a wide geographic range across Asia (Additional file 32: Table S1). On the basis of the nucleotide polymorphisms identified by Ho-LAMap, we could divide the sequences of the cultivated varieties into four haplotypes that were placed into two classes by phylogenetic analysis: haplotypes 1 and 3 in class A and haplotypes 2 and 4 in class B (Fig. 4a, b). Cultivars having class A haplotypes showed significantly higher *OsLG3* expression levels and a longer grain phenotype than those with class B haplotypes by qRT-PCR analysis (Fig. 4c, d). Furthermore, transient assays indicated that the four consensus SNPs, rather than indel1, played a significant role in regulating *OsLG3* expression differences between genotypes, resulting in diversity on grain length (Fig. 4a, e).

Geographically, 32 temperate *japonica* cultivars with Hap4 were from northern China, Russia, Korea, and Japan, or higher elevation zones (Fig. 4f). In contrast, samples with Hap1, including 133 *indica* and 10 *javanica* cultivars, were from South China, Southeast Asia, East Africa and South America. This group also included 19 glutinous temperate *japonica* from the Yunnan-Guizhou Plateau or mixtures. The 26 *O. rufipogon* samples with Hap1 were from Southeast Asia and south China, whereas the 8 Hap4 *O. rufipogon* samples originated from Guangxi province in China (Additional file 32: Table S1). These observations indicate that a north–south differentiation in *OsLG3* occurred between *indica* and *japonica* during rice domestication.

We analyzed the genetic diversity [34] in *OsLG3* and its flanking regions (~400 kb) to determine whether *OsLG3* had undergone artificial selection during domestication of *indica*. Clearly decreased nucleotide diversity was observed at the *OsLG3* locus in *indica*, but not in temperate or tropical *japonica* accessions (Fig. 4g). The overall average nucleotide diversity of the 20 kb *OsLG3* flanking regions was higher than that of *OsLG3* in *indica*, but also significantly lower than in wild rice (Fig. 4h). The diversity in an approximately 100-kb region around *OsLG3* in *indica* was lower than that in *japonica* and wild rice. These results suggest that the decreased diversity in *indica* resulted from selection.

To gain deeper insights into the evolution of *OsLG3*, we analyzed all polymorphic sites in the entire gene region of a larger population. We identified 7 haplotypes in 504 diverse cultivars (3 *indica* and 4 *japonica* haplotypes) and 13 haplotypes in 15 wild accessions. A minimum-spanning tree of haplotypes revealed two distinct clusters – separate cultivar and wild rice haplotype clusters – in *OsLG3*. The cultivars divided into two subgroups, *indica* and *japonica* (Fig. 4i). The wild accessions contained both large and small grain haplotypes. Only some *O. rufipogon* accessions from China had small grain haplotypes, which might have been inherited to ancient *japonica*. All *indica* haplotypes had large grain *OsLG3* haplotypes and one haplotype was present in two wild accessions, indicating the large grain haplotypes in *indica* might be derived from an *O. rufipogon* accession with a large grain haplotype. The large grain allele of *OsLG3* in *javanica* and some glutinous template *japonica* from the Yunnan-Guizhou Plateau or admixed accessions may be derived from *indica* sources (Fig. 4f, i). Our results implied that *OsLG3* alleles in *indica* and *japonica* independently originated from distinct ancestors, consistent with previous conclusions that the two subspecies were domesticated from distinct ancestral gene pools [35].

### Genetic interactions with other related genes and potential utilization in rice breeding improvement

**Fig. 5:**
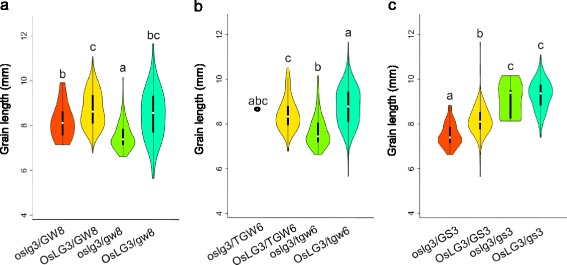
The genetic interactions between *OsLG3* and other grain length-related genes based on diverse germplasm. A box plot and a kernel density plot were generated as violin plots for different groups. **a**–**c** Relationship between *OsLG3* and other grain size-related genes, including *GS3* (**a**), *GW8* (**b**), and *TGW6* (**c**). The violin map was constructed in R. Different letters above columns indicate statistically significant differences between groups (Tukey’s honestly significant difference test, *P* < 0.05). Landraces, genotypes, and phenotypes are listed in Additional file 32: Table S1

**Fig. 6:**
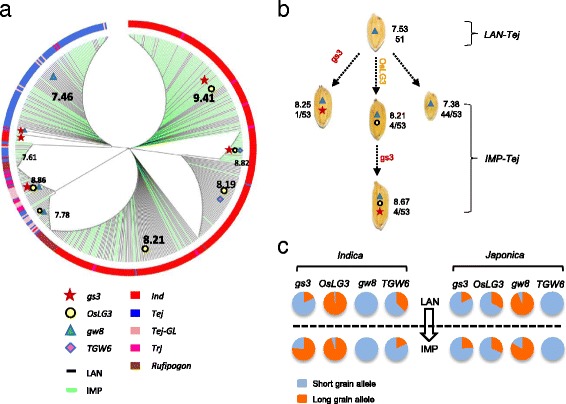
Breeding improvement of *OsLG3*. **a** Phylogenetic analysis of four grain length-related genes in 480 accessions. The phylogenetic tree of 480 varieties was constructed based on different functional SNPs or indels (listed in Additional file 32: Table S1) by MAGE 6.0. All varieties were categorized by allelic variations in the FNP of *OsLG3*, *GS3*, *GW8*, and *TGW6*. The red pentagram refers to the beneficial allele of *gs3*, yellow dot to the beneficial allele of *OsLG3*, blue triangle to the beneficial allele of *GW8*, purple prism to the beneficial allele of *TGW6*, black lines to landrace varieties and green lines to improved varieties. Different colors reflect the different subgroups, with its abbreviation as in Fig. 4. **b** Improvement of haplotype combination for four grain length-related genes, as in **a**. Top numbers indicate average grain length; bottom numbers correspond to accessions number with a type of haplotype combination in the subgroup. **c** Spectra of allele frequencies comparing landrace and improved varieties at the causal polymorphisms of *GS3*, *OsLG3*, *GW8*, and *TGW6* during modern breeding in the respective subgroups. *LAN* landraces, *IMP* improved varieties

To dissect the genetic interaction between *OsLG3* and other genes controlling grain length, we analyzed the promoter sequences of *OsLG3* and various haplotypes of *GS3*, *GW8*, *TGW6*, *GW2*, and *GL3.1* in 498 accessions (Additional file 32: Table S1, Additional file 33: Table S2). By re-sequencing our germplasm resources, we found that the alleles of *GW2* and *GL3.1* responsible for longer grains were quite rare. We then investigated *OsLG3*, *GS3*, *GW8*, and *TGW6* and analyzed the association between their grain length and allelic variants. All varieties were categorized by allelic variations in the functional nucleotide polymorphisms (FNPs) of *OsLG3*, *GS3*, *GW8*, and *TGW6*. The beneficial allele of *OsLG3* had an epistatic effect on grain length for *GW8* and *TGW6*; however, the beneficial allele of *GS3* had a recessive epistatic effect on grain length for *OsLG3* (Fig. 5a–c and Additional file 26: Figure S26). Our results suggest these loci cooperatively regulate grain length in rice, as accessions with the same haplotype for a given gene exhibited grain length diversity, and that variations at *OsLG3* and *GS3* are major factors affecting grain length diversity on a wide range of genetic backgrounds (Fig. 6a). Phylogenetic analysis showed that landrace varieties and improved varieties in the *indica* group were distinguished remarkably on account of improvement of grain length, although it fitted well within the *japonica* group. This indicated that grain length in *indica* rice has been widely improved by the presence of beneficial alleles of *GS3* and *OsLG3*, whereas there remains room for improvement for grain length in *japonica* (Fig. 6a, c and Additional file 27: Figure S27). In the *japonica* group, grain of some improved varieties was significantly longer because of introgression of the beneficial allele of *OsLG3* (Fig. 6a, b). Our results suggest that *OsLG3* has potential for improvement of grain length in *japonica*.

## Discussion

Grain size traits of rice are complex and generally controlled by multiple genes. Genetic control of these characteristics has been investigated over the last decade. To date, a total of 11 QTL for grain size (including *GS3*, *GL3.1*, *GW6a*, *TGW6*, *GL7*, *GLW7*, *GS5*, *GW5*, *GW8*, *GS2*, and *GW2*) have been cloned from natural rice varieties by a classic map-based approach. Despite these efforts, the mechanisms that establish the final size of grain remain poorly understood. In this research, we conducted GWAS for rice grain length based on high density SNPs (an average of 1 SNP per 40 bp) from 504 accessions and identified 99 QTL for grain length. This provides important information for cloning novel grain length genes in the future. Our findings confirmed that Ho-LAMap was able to mine genes rapidly over a wide range of experimental variables using deep-sequence data and that natural variations in the *OsLG3* promoter region conferred grain length and weight.

In rice, *GS3*, *GL3.1*, *GW2*, *GW5*, and *GW6a* controlled grain size by regulating cell proliferation, whereas *GLW7* did so by regulating cell expansion. *GS2*, *GS5*, *GL7/GW7*, and *GW8* influenced both cell division and cell expansion [36]. In this study, we reported a novel transcriptional regulatory factor that controlled grain length by influencing cell proliferation. Expression analysis showed that elevated expression levels of *OsLG3* increased the expression levels of many key genes involved in the cell cycle. The glumes from NIL(SLG) had the same cell length with NIP, but cell numbers of epidermal cells in the longitudinal direction in NIL(SLG) were more than those in Nipponbare. Thus, these findings indicate that *OsLG3* contributes to grain length by promoting cell division.

Many QTL related to rice grain size or weight were reported to enhance the grain yield. Among them, *GW2*, *GS2*, and *GW5* increased grain size and yield to a large extent; however, they caused a sudden increase in the percentage and degree of chalkiness, resulting in a sharp decrease in grain quality. Our results revealed that the introduction of SLG allele of *OsLG3* into existing elite *japonica* varieties might increase the grain length, weight and yield without disturbing the grain quality. According to the pedigree records, Yunguang8 is one of the first two-line hybrid rice most widely grown in Yunnan province of China because of its particularly high yield and good appearance quality. Its parents are the Yunhui11 and Nongken58S, a formerly widely used good-quality PTGMS line in China. A resequencing study showed that Yunguang8 carried the beneficial allele of *OsLG3* deriving from Yunhui11 and the *gw8* ^*Basmati*^ allele deriving from Nongken58S, respectively (Additional file 32: Table S1). Thus, the combination of the *OsLG3* ^*SLG*^ and *gw8* ^*Basmati*^ alleles provides a good example, which could have been employed by breeders, for simultaneously improving rice yield and grain quality over what is currently achievable.

Rice is a major cereal and a model system for plant biology, however, the origin of cultivated rice and its domestication history have long been debated. During the past decades, several models were proposed for rice domestication. Asian cultivated rice contains five genetically distinct ecotype groups, namely *indica*, *aus*, temperate *japonica*, tropical *japonica*, and *aromatic*. A simple single origin of cultivated rice suggested that the cultivated rice was primarily domesticated from the annual genotypes, and the two subspecies *indica* and *japonica* were regarded to be molded after domestication [37, 38]. Londo et al. [39] reported that *indica* and *japonica* were domesticated independently. Another hypothesis indicated that *japonica* rice was first domesticated and *indica* rice was subsequently developed from crosses between *japonica* rice and another clade of *O. rufipogon* [40]. Recently, Civáň et al. [41] suggested that there were three geographically separate domestications of Asian rice and concluded that rice domestication was a multiregional process separately producing the *indica*, *japonica*, and *aus* types of rice. Some well-characterized domestication genes (*Sh4*, *Prog1*, *Bh4*, and *qSH1*) in rice were found to be fixed in both subspecies with the same alleles, thus supporting the hypothesis of a single domestication origin [42–45]. However, our findings revealed that *OsLG3* alleles in *indica* and *japonica* independently originated from distinct ancestors (Fig. 4i). In view of the fact that grain size is a target trait for both domestication and artificial breeding, this conclusion was consistent with the hypothesis that *indica* and *japonica* were domesticated independently. Although rice is a self-crossing species, in this study, gene flow was detected from domesticated to two (out of the total 15) wild accessions (Fig. 4i). This is in agreement with a previous report of gene flow from domesticated to wild crop in another self-crossing species [46].

As demonstrated by our study, grain length is a complex trait associated with population structure in rice (Additional file 4: Figure S4a and Additional file 5: Figure S5). Although we detected two major QTL using the CMLM model (Additional file 34: Table S5), a large amount of middle effect and minor effect QTL, even many other major QTL for grain length (e.g., *GW2*, *OsLG3*, *TGW6*, *gw8*), could not be identified (Additional file 3: Figure S3). This indicates that not all associations that are eliminated in the CMLM model are false. GWAS incorporating Q and K in a CMLM controls *P* value inflation well, but leads to false negatives, missing some potentially important true associations. It is possibly due to the confounding between the covariates and test marker weakening the signals of Quantitative Trait Nucleotides (QTNs), resulting in false negatives in CMLM [47]. Recently, Liu et al. [47] proposed a new method, FarmCPU (Fixed and random model Circulating Probability Unification), to control false positives as well as CMLM with reduction in false negatives. FarmCPU iteratively performs marker tests with pseudo QTNs as covariates in a fixed effect model and optimization on pseudo QTNs in a random effect model. To some extent, this process removes the confounding between testing markers and kinship, prevents model over-fitting, and controls false positives simultaneously. We reanalyzed our data using FarmCPU. Seven, four, and two QTL were identified by FarmCPU, including the first three PCs, two PCs, and one PC as covariates, respectively; further, these QTL hit two (*GS3* and *SSG6*), two (*GS3* and *GW6a*), and one (*GS3*) known genes, respectively (Additional file 28: Figure S28), and overlapped with five, three, and one published QTL (Additional file 38: Table S15). When using FarmCPU including the first three PCs, we detected five and five QTL in the *indica* and *japonica* subpopulations, respectively, and these QTL hit two (*GS3* and *GW5*) and one (*TGW6*) cloned genes, respectively (Additional file 29: Figure S29), overlapping with four and two of the published QTL (Additional file 38: Table S15). FarmCPU outperformed CMLM with respect to controlling inflation of *P* values (Additional file 28: Figure S28e–h), identifying new QTL for rice grain length, and overlapping with known loci (Additional file 28: Figure S28a–d and Additional file 34: Table S5 and Additional file 38: Table S15). Unlike the FarmCPU, our association analysis for the rice grain size traits in Ho-LAMap employed GLM (Q) to try to avoid false negatives. There is no doubt that FarmCPU also could be integrated with Ho-LAMap in identifying new QTL because of its improved statistical power.

The era of deep-sequencing of vast arrays of germplasm has arrived and DNA sequencing has become quicker and cheaper. Thousands of rice accessions have been deep-sequenced [23] and thousands of QTL on grain size (also other agronomic traits) have been mapped in rice (http://archive.gramene.org/db/qtl/qtl_display?trait_category=Yield). However, how to rapidly identify the genes associated with agronomic traits utilizing these large amounts of sequence data and the linkage mapped QTL information in rice remains a challenge.

Ho-LAMap is an attractive method for rapid, cost-effective identification of natural variations underlying QTL based on co-localization in multiple populations (Additional file 30: Figure S30 and Additional file 33: Notes S3). Coupled parents of populations having different genetic backgrounds can be divided into two classes similar to generating contrasting genetic pools for analysis of individual traits. Using the Ho index, we effectively distinguish a large number of background interference signals (i.e., false positives) from all (Additional file 12: Figure S12). Furthermore, candidate region association mapping conducted on SNPs located in the overlapping intervals of targeted QTL avoids false-positive loci located outside the critical region.

Deep sequencing of more than 3000 core rice collections with very broad genetic diversity and collected worldwide has been accomplished and our team has completed construction of approximately 100 recombinant inbred line populations based on accessions from our MCC panel. We expect that Ho-LAMap will facilitate gene isolation and rice breeding through molecular design by reducing the time for the construction of large NIL-F_2_ populations and fine mapping of QTL thus avoiding extremely laborious, time-consuming, and expensive fieldwork required for identification and cloning of agronomically important genes (Additional file 31: Figure S31) such as those for stress resistance.

## Methods

### Field planting and measurement of grain and yield traits

Rice plants were examined under natural field conditions at the two Experimental Stations of China Agricultural University, Beijing and Hainan, China. The planting density was 13.3 cm between plants in rows that were 23.3 cm apart. Field management, including irrigation, fertilizer application, and pest control, followed normal agricultural practices. Harvested rice grains were air-dried and stored at room temperature for at least 3 months before testing. Fully filled grains were used for measuring grain width, length, and weight. Ten randomly chosen grains from each plant were lined up width-wise along a Vernier caliper to measure grain width and then arranged length-wise to measure grain length. Grain weight was based on 200 grains and converted to 1000-grain weight.

### Plant material and backcross populations

The extra large-grain rice accession SLG-1 (*Oryza sativa* L*.* ssp. *japonica*) and another two large-grain varieties, Haobuka and IRAT109, were selected from more than 7000 germplasm and used as desirable donor parents. The smallest grain accession, Chuanqi (ssp. *indica*), and two medium grain accessions, Nipponbare and Yuefu (ssp. *japonica*), were selected as recurrent parents. Four hybrid combinations (BILs from Nipponbare and SLG-1; CSSLs from Yuefu and IRAT109; BC_1_F_2_ from Chuanqi and SLG-1; and BC_1_F_2_ from Nippobare and Haobuka) were created by the six varieties.

The Nipponbare x SLG-1 cross: Four BC_4_F_2_ populations for primary QTL mapping was constructed through selective backcrossing of lines which had large grain at each backcross generation. Three BC_4_F_3_ populations for further QTL mapping were derived from some BC_4_F_2_ plants without the known large-effect QTL (*gs3* and *GW2*). Using a similar strategy, we developed another two BC_1_F_2_ populations: one from SLG-1 crossed with Chuanqi, the other one from the cross between Nipponbare and Haobuka.

The Yuefu x IRAT109 cross: Yuefu was crossed with IRAT109 and backcrossed with Yuefu for five generations, before selfing to produce BC_5_F_3_ plants that were genotyped using 176 simple sequence repeat markers evenly distributed across all 12 rice chromosomes. Finally, we generated a fixed population of 271 CSSLs, with each containing an average three chromosome segments from the donor in the Yuefu genetic background.

### Selection of germplasm, sequencing and SNP identification

The 506 worldwide accessions (Additional file 32: Table S1) contributed by the Chinese Academy of Agricultural Sciences included a mini-core collection of 248 accessions selected from a core collection of 932 accessions established from 61,470 *O. sativa* accessions preserved in the China National Crop Gene Bank [25], and 256 accessions selected based on isozyme diversity [48], used as parental lines in the international rice molecular breeding network, plus two accessions with the largest and smallest grains, respectively.

Genomic DNA was prepared from bulk-harvested leaves of a single young plant for each sampled accession by a modified CTAB method either at the International Rice Research Institute or at the Chinese Academy of Agricultural Sciences. Genomic DNA samples were then shipped to BGI-Shenzhen and were used to construct Illumina index libraries following the manufacturer’s protocol. Following quality control, at least 3 μg of genomic DNA of each sample was randomly fragmented by sonication and size-fractionated by electrophoresis. DNA fragments of approximately 500 bp were purified from each of 24 accessions and were labeled independently using distinct 6 bp nucleotide multiplex identifiers, followed by pooling prior to library construction for next generation sequencing. Each sequencing library was sequenced in six or more lanes on the HiSeq2000 platform and 90-bp paired-end reads were generated. Subsequently, the reads from each sample were extracted based on their unique nucleotide multiplex identifiers as 83 bp reads (90 – 6 – 1, where 1 is the ligation base “T”). To ensure high quality, raw data were filtered by deleting reads having adapter contamination or containing more than 50% low quality bases (quality value ≤ 5).

The SNP data utilized for GWAS were generated as described in ‘The 3000 rice genomes project’ [23], and the SNP identification process was as follows:

- SNP calling for each sample were performed independently using the UnifiedGenotyper package in GATK with a minimum phred-scaled confidence threshold of 50, and a minimum phred-scaled confidence threshold for emitting variants at 10. To ensure the quality of variant calling, the conditions for every site in a genome were set at > 20 for mapping quality, > 50 for variant quality, and > 2 for the number of supporting reads for every base.
- Using IRGSP-1.0 as the reference, the 3000 sequenced genomes had an average depth of approximately 14×, ranging from approximately 4× to greater than 60×, and yielded a combined total of approximately 17 TB of high quality sequence data. Of the 3000 entries, 504 accessions used in this study had an average sequence depth of 14.9×. Burrow–Wheeler alignment followed by variant calling using GATK identified approximately 10 million SNPs.
- The SNPs finally identified in this study were 3,585,228 controlled by MAF ≥ 5% and missing rates ≤ 30%.

### GWAS studies and candidate-region association mapping

GWAS of *indica*, *japonica*, and the full population were conducted using their corresponding genotype datasets. In each panel, only the SNPs with MAF > 5% were used for association analyses. Three models – the naive model, GLM (Q), and CMLM – were used for association analysis [17]. The Q matrix and K matrix were estimated by STRUCTURE 2.0 and GAPIT function in R [49], respectively, and the R version relies on the version of EMMA.

### QTL mapping and fine mapping

A genetic map was constructed using MapMaker3.0/EXP version 3.0 [50]. QTL analysis was performed by IciMapping3.1 [51] along with the composite interval mapping method, and the threshold was obtained by 1000 permutations. The substitution mapping strategy was used for fine mapping [52].

### Simulation analysis

The significant SNPs within the QTL regions on both sides of physical position of each of seven well-characterized genes (i.e., *GS3*, *GS5*, *GW5*, *GW8*, *OsLG3*, *GL7*, and *TGW6*) and phenotypes of grain length, grain width, and grain weight were used in our simulation studies. The parents of biparental populations used for QTL mapping were randomly selected from the two tails of the population by extreme trait performance. Since there are two alleles for each SNP, say *A* and *a*, let *p*_*H*_ denote the allele frequency of *A* in one tail of the population with high trait performance, and *p*_*L*_ denote the allele frequency of *A* in the other tail with low trait performance. That is to say, the allele frequency of *A* for one parent was *p*_*H*_, and that for the other parent was *p*_*L*_. If the SNP locus is polymorphic between two parents, it could be significantly associated with the trait of interest. The power of Ho-LAMP method can be estimated as *p*_*H*_ (1 – *p*_*L*_) + (1 – *p*_*H*_) *p*_*L*_. To evaluate the effect of population structure on the power of Ho-LAMP method, we conducted simulation experiments for all 504 rice cultivars, *Japonica* cultivars, and *Indica* cultivars as groups. We also tried percentages of 0.05, 0.10, and 0.15 to select sub-populations with extreme phenotypic performance on both sides. Results were largely similar; therefore, 0.05 was used as the percentage cut-off to determine the size of the two tails of the population.

Simulation of cross number also used significant SNPs of some of these well-characterized genes (i.e., *GW8*, *GS3*, and *TGW6*). Firstly, 504 accessions were divided into two parts by functional alleles of each gene, called A and a. Randomly selected similar numbers of varieties from each part were used to calculate the Ho-index for functional alleles in the candidate region. We simulated 1000 repeats and counted the times when only the functional allele had the maximum Ho-index, denoted as one time and three replicates for each gene. We evaluated the power as the proportion of detecting the target gene successfully in 1000 replications of simulation. Finally, we set different cross numbers [2–50] to carry out the simulation and defined the ideal number of crosses for each gene.” Because the numbers are not citations. The same process was applied to the *indica* and *japonica* populations, respectively.

### Vector construction and plant transformation

Full-length cDNA of *OsLG3* was amplified from the first-strand cDNA of IRAT109 with specific primers to generate the overexpression construct (Additional file 33: Table S17) using PrimeSTAR HS DNA Polymerase (TaKaRa). The sequence-confirmed PCR fragment was digested with *Kpn* I and *Pac* I, and inserted into the vector pMDC32 under the control of the *Cauliflower Mosaic virus* (CaMV) 35S promoter.

To generate the RNA interference (RNAi) construct, a fragment targeting the 294 bp coding region of *OsLG3* was amplified with specific primers P3 and P4, digested with *Kpn* I and *Bam*H I and then *Spe* I and *Sac* I sites, and subsequently inserted into the pTCK303 vector as previously described [53]. Primers used in these experiments are listed in Additional file 33: Table S17. To generate transgenic plants, the constructs were transformed into Nipponbare by *Agrobacterium*-mediated transformation [54]. Positive transgenic plants were selected by germinating transgenic seeds on 1/2 MS medium containing 50 mg/L hygromycin (Roche, Germany).

### RNA extraction and expression analysis

For expression analysis, rice leaves were flash-frozen in liquid nitrogen, total RNAs were extracted using RNAiso Plus (TaKaRa) according to the manufacturer’s instructions; 4 μg of the DNase-treated RNA were reverse transcribed using M-MLV reverse transcriptase (TaKaRa). The resulting cDNA samples were diluted five times and used as templates for PCR.

qRT-PCR was performed in an Applied Biosystems 7500 Real Time PCR system (ABI, USA) using SYBR Premix Ex Taq™ II (TaKaRa) as previously described [55, 56]. The gene-specific primers used for real-time PCR are listed in Additional file 33: Table S16. qRT-PCR was performed in triplicate for each sample, and the *Actin1* gene was used as the internal reference for data normalization using the 2^–ΔΔt^ method [57].

### Sub-cellular localization of *OsLG3* and transactivation activity assay

To generate the 35S::*OsLG3*-GFP construct, the full-length ORF of *OsLG3* without the terminal codon was amplified with the corresponding primers (Additional file 33: Table S17). The amplified fragments were digested with Kpn I and Pac I, and then cloned into the pMDC83 vector and fused with the GFP reporter gene driven by the CaMV 35S promoter. The recombinant constructs of the 35S:*OsLG3*-GFP fusion and GFP alone were transiently transfected into onion epidermal cells, using a PDS-1000/He system (Bio-Rad, USA) at 1100 psi. After incubation at 25 °C for 24 h, the fluorescence signal was examined through a confocal laser scanning microscope FV1000 (Olympus, USA) with excitation at 488 nm and emission at 525 nm.

For the transactivation assay, plasmids pGBKT7-*OsLG3* (full length coding region of *OsLG3*), pGBKT7-*OsLG3*-N (N-terminal of *OsLG3*, amino acids 1–108), pGBKT7-*OsLG3*-C (C-terminal of *OsLG3*, amino acids 109–209) were constructed. The pGBKT7 vector was used as a negative control and pGBKT7-53 was used as a positive control. These constructs were introduced into yeast strain AH109 by LiAc-mediated yeast transformations, and screened on selective medium plates without tryptophan (SD/–Trp). The PCR-verified transformants were transferred to SD medium without tryptophan/histidine/adenine (SD/–Trp/–His/–Ade) for 3 days. Transactivation activities were performed according to the in vivo agar plate assay (x-α-gal in medium).

### Histological analysis

Milled rice grains for scanning electron microscopy were transversely cut in the middle with a knife and coated with gold under vacuum conditions. The morphology of starch granules in the belly part of the endosperm was examined with a scanning electron microscope (Hitachi, S-570, China Atomic Energy Research Institute) at an accelerating voltage of 12 kV. The analysis was based on at least three biological replications of mounted specimens. All procedures were carried out according to the manufacturer’s protocol.

### Nucleotide diversity analysis and minimum spanning tree

The average nucleotide diversities (π) of *indica*, *temperate japonica*, *tropical japonica*, and wild rice subpopulations were estimated in non-overlapping 10 kb windows using an in-house Perl script; missing data positions were included, with a modification of population size [58].

Five hundred and four diverse cultivated rice and 15 *O. rufipogon* accessions from around the world (Additional file 32: Table S1) were used to construct a minimum spanning tree for *OsLG3*. Arlequin version 3.5 [59] was used to define the haplotypes and calculate the minimum spanning tree among haplotypes. Arlequin’s distance matrix output was used in Hapstar-0.7 [60] to draw a minimum spanning tree.

### Phylogenetic and genetic interaction analysis

The phylogenetic tree of 480 varieties was constructed based on SNPs and indels that were proven FNPs (listed in Additional file 32: Table S1) by MAGE 6.0. The EvolView [61] online tool was used for visualizing the phylogenetic tree. Grain length was measured by Vernier calipers and the violin map for genetic interaction analysis was constructed in R. Multiple comparisons were made by Tukey’ honest significant difference in R. Landraces and raw data are listed in Additional file 32: Table S1.

## Additional files

Additional file 1: Figure S1. Geographic origins of 504 rice accessions. (a) Geographic origins of worldwide rice cultivars. The smaller pie charts on the world map correspond to the country-specific distribution of subpopulations sampled (note: China was divided into several major rice growing regions). The size of the pie chart is proportional to the sample size and colors within each pie chart are reflective of the percentage of samples in each subpopulation. Seeds representing each subpopulation are displayed with and without hull in the center, with 5 mm scale bar. (b) Geographic distribution of these accessions. Number of countries sampled in each geographical region are indicated on top of the bars. (PDF 250 kb)

Additional file 2: Figure S2. Frequency distribution of grain length in the mini core collection (MCC population). (PDF 117 kb)

Additional file 3: Figure S3. Comparison of GWAS of grain length in the full population using LM model (a), CMLM model (b), and GLM (Q) model (c). In these Manhattan plots, for the significant loci identified, known loci are shown in red. Blue horizontal solid lines indicate the genome-wide significance threshold. (PDF 114 kb)

Additional file 4: Figure S4. Correlation between grain length and population structure in the natural population and frequency distribution between two genotypes in subpopulation about *GS3*, *GS5*, *TGW6*, *GW8*, and *OsLG3*. (a) Distribution of germplasms with different grain length according to PC structure. (b) Frequency distribution between two genotypes in subpopulation about *GS3*, *GS5*, *TGW6*, *GW8*, and *OsLG3*. The rate of each haplotype under two sub population divided by Q for these genes. *Ind*, *indica; jap*, *japonica*. (PDF 246 kb)

Additional file 5: Figure S5. Effect of Q structure (*indica* and *japonica*) for grain length. (a) Comparison of grain length between big-grain-haplotype and small-grain-haplotype when Q structure exists. (b) Comparison of grain length between big-grain-haplotype and small-grain-haplotype when the phenotype variation from Q structure was removed. *Ind*, *indica*; *jap*, *japonica*. Data are means ± SEM. Letters indicate a significant difference at *P* < 0.01 (*n* = 3) by the Student’s *t*-test. (PPTX 362 kb)

Additional file 6: Figure S6. Size of association regions about *GS3*, *TGW6*, *GW8*, and *OsLG3*. Manhattan plots in candidate region of three known genes and *OsLG3* in different models. The red points indicate their SNPs within gene region, respectively. The horizontal full lines indicate the genome-wide significance threshold (0.05/n). (PDF 311 kb)

Additional file 7: Figure S7. Grains from six parents. Scale bar, 5 mm. (PDF 109 kb)

Additional file 8: Figure S8. Frequency distribution of variation of grain traits in biparental populations derived from NIP and SLG. (a–d) Phenotype variants of grain traits for populations 07DH010, 07DH011, 07DH013, and 07DH014, respectively. (PDF 103 kb)

Additional file 9: Figure S9. QTL detected in the four crosses derived from six varieties. (a) Distribution of grain shape QTL and genes on genetic linkage map. Blue strip refers to grain length QTL, green to 1000-grain weight and grain width QTL, red to grain thickness QTL and arrows to cloned genes. (b) Heat map for effect of grain length QTL mapped by four linkage populations. Rows of the heat map correspond to the 14 QTL for grain length. NIP, Nipponbare; SLG, SLG-1; CQ, Chuanqi; YF, Yuefu; IR109, IRAT109; HBK, Haobuka. (PDF 224 kb)

Additional file 10: Figure S10. Fine mapping of *qGL3-3*. White bars represent chromosomal segments for NIP homozygote, black for SLG homozygote, and grille for heterozygote, respectively. Progeny testing was used to confirm the genotypes at the *qGL3-3* locus. S, segregation; D, desegregation. (PDF 78 kb)

Additional file 11: Figure S11. Correlations between the grain traits in the MCC panel (a) and 07DH014 population (b). Number in blue, phenotypic correlation in r^2^ between traits. (PDF 124 kb)

Additional file 12: Figure S12. Simplified scheme for application of Ho-LAMap to rice. (a) We cross diverse founder varieties (i.e., variety that is significantly different to the reference parent on grain traits) with reference parent (usually has small grain). The founder varieties are deep sequenced by the second-generation sequencing platforms, as in Additional file 30: Figure S30. In several crosses that have detected targeted QTL, the majority of SNPs between the QTL interval will segregate in a 1:1 founder varieties:reference parent ratio. However, the SNP responsible for the change of phenotype is the same in all founder parents, which can detect the targeted QTL. If we define the Ho (observed heterozygosity per locus) index as the ratio between the number of heterozygous crosses corresponding to each SNP locus and the total number of crosses which have detected targeted QTL, we expect this index would equal 1 near the causal SNP and 0.5 for the unlinked loci. (b, d) Candidate region association mapping. The brown horizontal dashed lines indicate the genome-wide significance threshold. The red points indicate significant loci within candidate gene. (c) Ho index plots for the target QTL. Red regression lines were obtained by averaging SNP indices from a sliding window analysis. (PDF 105 kb)

Additional file 13: Figure S13. Simulation reveals effectiveness of Ho-LAMap in different subgroups for several known genes (such as *GW8*, *TGW6*, etc.) about grain size when using Ho-LAMap. The subgroups contain *indica* and *japonica* population. (PPTX 671 kb)

Additional file 14: Figure S14. Simulation reveals the cross number needed for three known genes for grain size when using Ho-LAMap. Simulation of crosses number also used significant SNPs of some of these well characterized genes (i.e., *TGW6* (a), *GS3* (b), and *GW8* (c)). The x-axis value indicates cross number. Full pop, the full population; *indica* and *japonica*, the *indica* and *japonica* subgroup. We evaluated the power as the probability of detecting the target gene successfully in 1000 replications of the simulation. (PDF 227 kb)

Additional file 15: Figure S15. Identification of the causal SNPs of QTL *qGL3-2* using Ho-LAMap. We also used Ho-LAMap to clone *OsLG3b* from *qGL3-2*, a new gene for grain length, which encodes a MADS-box transcription factor. The top is Manhattan plot for candidate region association mapping for QTL region of *qGL3-2*; the middle correspond to Ho index plots for QTL region of *qGL3-2*. The bottom correspond to candidate gene (*OsLG3b*), the green dashed lines label the region for significant signal. (PDF 82 kb)

Additional file 16: Figure S16. Nucleotide diversity analysis for the promoter region and CDS of *OsLG3*. (PDF 59 kb)

Additional file 17: Figure S17. *OsLG3* expression levels in organs from NIP and NIL(SLG) plants. FL, flag leaf at the heading date; S, stem; H4B and H2B, hulls at 4 and 2 days before heading; H4A, hull at 4 days after heading; 5E and 10E, endosperm at 5 and 10 days after fertilization; *n* = 3. Data are given as mean ± SEM. (PDF 84 kb)

Additional file 18: Figure S18. Phenotypes of grain in three *OsLG3*-overexpressing lines and three RNAi-*OsLG3* lines. (a, d) Grain length of the transgenic and control lines (*n* = 30). (b, e) 1000-grain weight in the transgenic and control lines (*n* = 30). (c, f) Relative expression levels of *OsLG3* in young panicles of the transgenic and control lines were detected by qPCR, with data normalized to *OsActin1* levels (*n* = 3). All data in c, f–h, and j–l are presented as means ± SEM. **P < 0.01, Student’s *t* test. (PDF 77 kb)

Additional file 19: Figure S19. A field trial of NIP and NIL(SLG) plants. (a) The morphology of the NIL plants. Scale bar, 10 cm. (b) Plant height. (c) Tiller number. (d) Number of grains per panicle. (e) Heading date. All phenotypic data in b–e were measured from plants grown with 20 × 20 cm spacing in paddies under normal cultivation conditions. Data represent mean ± SEM. (*n* = 30). Student’s *t* tests were used to generate *P* values. (PDF 111 kb)

Additional file 20: Figure S20. *OsLG3* regulates grain length by changing cell division patterns. (a) The grains of NIP and NIL(SLG) plants (top) and scanning electron microscope images of the outer glume (middle) and inner epidermal cells of the lemma (bottom) before anthesis. Scale bars, 5 mm (white; top) and 300 μm (yellow; middle and bottom). (b) Cell length of outer epidermal cells in the longitudinal direction in a (*n* = 12). (c) Cell width of outer epidermal cells in the longitudinal direction in (a) (*n* = 12). (d) Total cell number of outer epidermal cells in the longitudinal direction. (e) Cell length of inner epidermal cells in the longitudinal direction in (a) (*n* = 12). (f) Cell width of inner epidermal cells in the longitudinal direction in (a) (*n* = 12). (g) Total cell number of inner epidermal cells in the longitudinal direction. All data represent means ± SEM. *P < 0.05, **P < 0.01, Student’s *t* test. (PDF 134 kb)

Additional file 21: Figure S21. The effect of *OsLG3* on the expression of genes involved in cell cycle. The expression analysis was conducted using 7-cm long young panicles. *OsActin1* was used as the control and the values of expression levels in Nipponbare were set to 1 (*n* = 3). Data are given as mean ± SEM. Student’s *t*-test was used to generate the *P* values; * *P* < 0.05, ** *P* < 0.01, respectively. (PDF 135 kb)

Additional file 22: Figure S22. *OsLG3* does not affect grain quality. (a) Scanning electron microscopy images are transverse sections of starch granule of NIP and NIL(SLG). Scale bars, 50 μm (green line). (b) Comparison of brown grains NIP and NIL(SLG). Scale bar, 2 cm. (c) The ratio of grain length to width (*n* = 30). (d) Percentage of grain with chalkiness (%) (*n* = 6). (e) Square of chalky endosperm (%) (*n* = 6). Data are given as means ± SEM. Student’s *t*-test was used to generate the *P* values; * *P* < 0.05, ** *P* < 0.01, respectively. (PPTX 405 kb)

Additional file 23: Figure S23. Overexpression of *OsLG3* in NIP has large effect on grain length and cell number. (a) The mature rice grain images of NIP (left) and over-expression lines (right), OE-3, OE-4, and OE-5, respectively. The scanning electron microscope images are the outer glume epidermal cells of the lemma from the spikelet hulls of NIP (left) and over-expression plant (right) before anthesis, respectively. Scale bars, 5.0 mm (white line) and 300 μm (yellow line), respectively. (b) Grain length. (c–e) Cell length of outer epidermal cells in the longitudinal direction in a (*n* = 12). (f–h) Total cell number of outer epidermal cells in the longitudinal direction (*n* = 12). All phenotypic data in b–h were measured from plants grown with 15 × 20 cm spacing in paddies under normal cultivation conditions. All data represent means ± SEM. *P < 0.05, **P < 0.01, Student’s *t* test. (PDF 107 kb)

Additional file 24: Figure S24. Effect of RNAi-*OsLG3* in NIP on grain length. RNAi-*OsLG3* in NIP has large effect on grain length. (a) The mature rice grain images are control (left) and RNAi-1, RNAi-2, and RNAi-3, respectively (right). The scanning electron microscope images are the outer glume epidermal cells of the lemma from the spikelet hulls of NIP (left) and RNAi-*OsLG3* plant (right) before anthesis, respectively. Scale bars, 5.0 mm (white line) and 300 μm (yellow line), respectively. (b) Grain length. (c–e) Cell length of outer epidermal cells in the longitudinal direction in a (*n* = 12). (f–h) Total cell number of outer epidermal cells in the longitudinal direction (*n* = 12). All phenotypic data in b–h were measured from plants grown with 15 × 20 cm spacing in paddies under normal cultivation conditions. All data represent means ± SEM. *P < 0.05, **P < 0.01, Student’s *t* test. (PDF 89 kb)

Additional file 25: Figure S25. *OsLG3* expression activity was monitored by *pOsLG3:GUS* transgene expression. Histochemical analysis of GUS activity in root (a), stem (b), sheath (c), leaf (d), and the developing panicles (e). Scale bar, 1 cm. (PDF 90 kb)

Additional file 26: Figure S26. Genetic interactions among *OsLG3*, *GS3*, *GW8*, and *TGW6*. (a) Varieties were categorized by allelic variations of *OsLG3*, *GS3*, and *TGW6*. (b) Varieties were categorized by allelic variations in the functional SNP of *OsLG3*, *GS3*, and *GW8*. Grain length was measured by Vernier calipers, and the violin map was constructed in R. Multiple comparisons were done by Tukey’ HSD in R. Landraces and raw data were listed in Additional file 32: Table S1. (PDF 143 kb)

Additional file 27: Figure S27. The spectrum of allele frequencies between landrace and improved varieties at the causal polymorphisms of *GS3*, *OsLG3*, *GW8*, and *TGW6* during modern breeding in full population (a) and *japonica* subgroup (b). LAN, landrace; IMP, improved variety. (PDF 199 kb)

Additional file 28: Figure S28. Comparison of GWAS of grain length in the full population using MLM (a), FarmCPU included the first three PCs (b), two PCs (c), and one PC (d) as covariates. In these Manhattan plots, for the significant loci identified, five SNPs within a 200-kb range of known genes are marked as significant loci re-identified. Green horizontal solid lines in (b), (c), and (d) indicate the Bonferroni-corrected significant threshold (0.01/n). However, the genome-wide significance threshold of all methods in this study adopts the Bonferroni-corrected threshold with 0.05 (0.05/n) (see Additional file 38: Table S15). Quantile-quantile plots of these models are shown in e, f, g, and h, respectively. (PDF 261 kb)

Additional file 29: Figure S29. Comparison of GWAS of grain length in the full population (a), *indica* (b), and *japonica* (c) subpopulations using FarmCPU included the first three PCs as covariates. In these Manhattan plots, for the significant loci identified, five SNPs within a 200-kb range of known genes are marked as significant loci re-identified. Green horizontal solid lines in (a), (b), and (c) indicate the Bonferroni-corrected significant threshold (0.01/n). However, the genome-wide significance threshold of all methods in this study adopts the Bonferroni-corrected threshold with 0.05 (0.05/n) (see Additional file 38: Table S15). Quantile-quantile plots in these three populations are shown in d, e, and f, respectively. (PDF 251 kb)

Additional file 30: Figure S30. Proposed strategy of gene discovery by Ho-LAMap. The number 1 and 2 in the red cycle indicate two pathways for gene discovery by Ho-LAMap. At the first way, a lot of QTL had been co-localized in many crosses by predecessors. Therefore, we re-sequenced parents of those crosses and isolated gene from the target QTL via Ho-LAMap. For the second way, we obtained the QTL region by GWAS and selected extreme materials to construct several F_2_ or recombinant inbred line populations according to peak SNP and phenotypes. Then, we could ascertain several crosses that can map the same QTL by linkage mapping and rapidly isolated gene from QTL by Ho-LAMap directly. (PDF 19 kb)

Additional file 31: Figure S31. Identification of the causal SNPs of QTL *sd1* using Ho-LAMap. The top is Manhattan plot for candidate region association mapping for QTL region of *sd1*; the middle corresponds to Ho index plots for QTL region of *sd1*. The bottom corresponds to candidate gene (*sd1*), the green dashed lines label the region for significant signal. (PDF 175 kb)

Additional file 32: Table S1. Information of *Oryza sativa* L. varieties and wild rice on variety name, geographic source, stratification referred by STRUCTURE, the integrated stratification, grain lengths, and allelic variations of grain length-related genes. (XLSX 115 kb)

Additional file 33: **Notes S1**. Natural population and linkage mapping (Additional file 2: Figure S2 and Additional file 11). **Notes S2**. Fine mapping of *qGL3-3* (Additional file 10). ** Notes S3**. Ho-LAMap – a potential method for validating association peak. **Table S2.** Summary of the taxa and source of 506 varieties of *Oryza sativa* L. **Table S3.** Environments used to evaluate association and linkage populations. **Table S4.** The heritability of grain traits in MCC1 Panel. **Table S9.** Summary of causal allele and type of variation about several grain size genes. **Table S10.** Grain phenotypes of six different parents. **Table S11.** Quantitative trait loci mapped in the four populations. **Table S12.** Simulation reveals the cross number need for several known genes (such as *TGW6*, *GS3*) about grain size when using Ho-LAMap. **Table S13.** *OsLG3* polymorphisms associated with grain length in the MCC panel. **Table S14.** Comparison of polymorphisms between three large grain parents and three small grain parents. **Table S16.** Primers used for fine mapping and sequencing. **Table S17.** Primers used for DNA constructs and transcript analysis. (DOCX 73 kb)

Additional file 34: Table S5. Genome-wide significantly associated loci (–log_10_ (P) ≥ 7.9) with GL in full population, *indica* and *japonica* population using CMLM model and QTL co-localized with linkage mapping. (XLSX 9 kb)

Additional file 35: Table S6. Genome-wide significantly associated loci with GL in full population using GLM (Q) model and QTL co-localized with linkage mapping. (XLSX 15 kb)

Additional file 36: Table S7. Genome-wide significantly associated loci with GL in *indica* population using GLM (Q) model and QTL co-localized with linkage mapping. (XLSX 11 kb)

Additional file 37: Table S8. Genome-wide significantly associated loci with GL in *japonica* population using GLM (Q) model and QTL co-localized with linkage mapping. (XLSX 14 kb)

Additional file 38: Table S15. Genome-wide significantly associated loci with GL in full population, *indica* population, and *japonica* population using FarmCPU and QTL co-localized with linkage mapping. (XLSX 13 kb)

Additional file 39: Supporting data. (XLSX 79 kb)
