# Supplementary figures and images for: OsLG3 contributing to rice grain length and yield was mined by Ho-LAMap
Source: BMC Biol. 2017 Apr 6;15:28. doi: 10.1186/s12915-017-0365-7 (PMC5383996; doi:10.1186/s12915-017-0365-7)

a

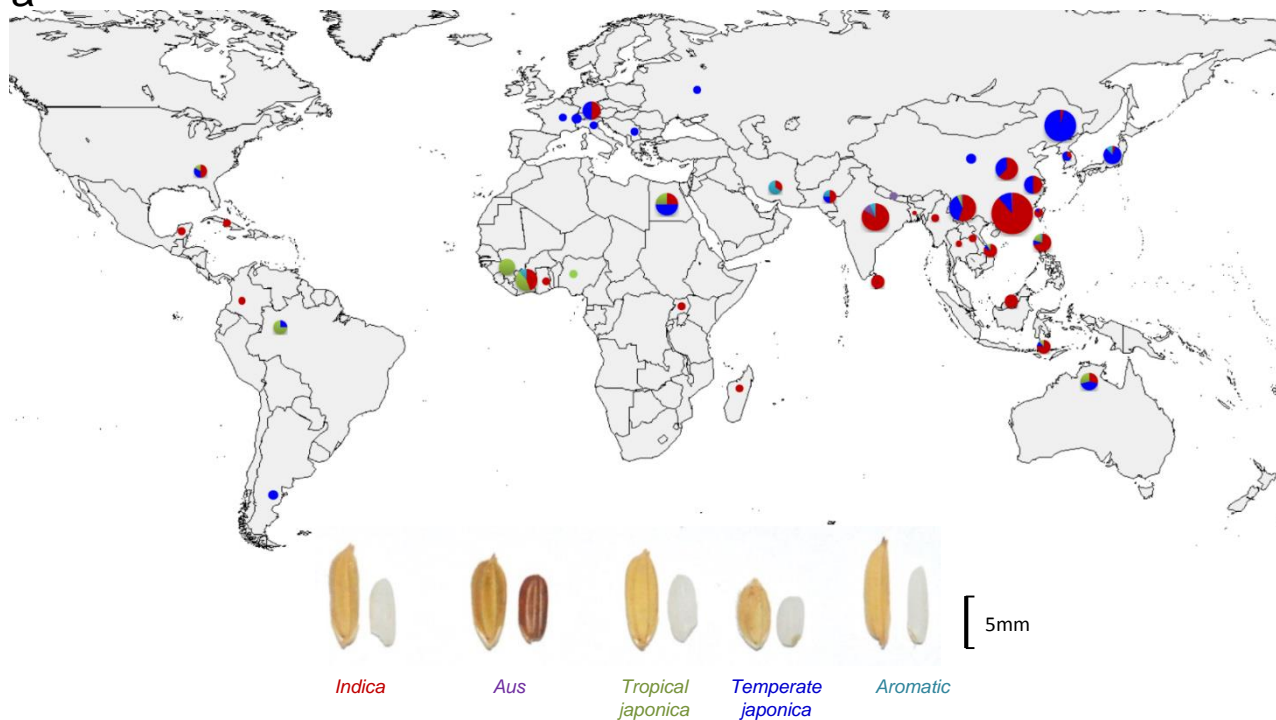

b

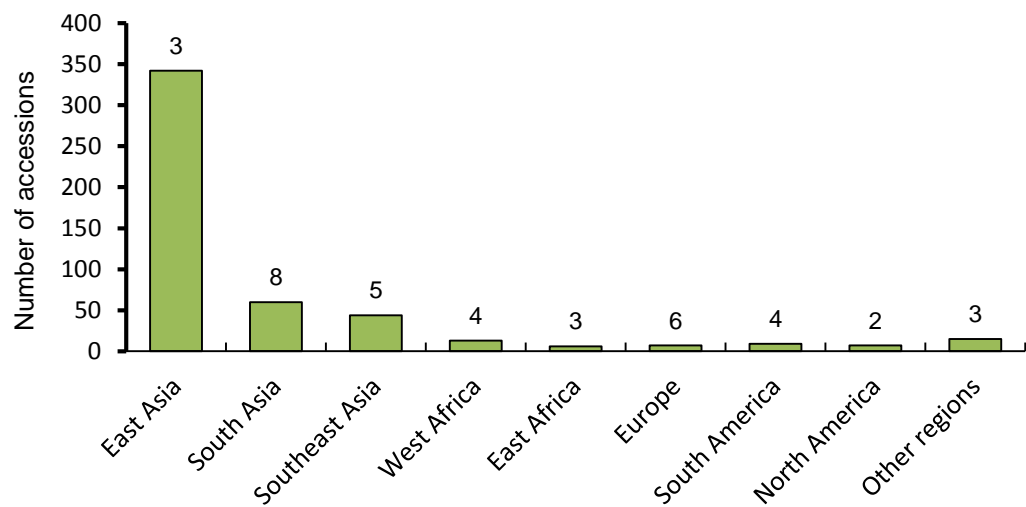

Supplement: Supplementary file 1 — Geographic origins of 504 rice accessions. (a) Geographic origins of worldwide rice cultivars. The smaller pie charts on the world map correspond to the country-specific distribution of subpopulations sampled (note: China was divided into several major rice growing regions). The size of the pie chart is proportional to the sample size and colors within each pie chart are reflective of the percentage of samples in each subpopulation. Seeds representing each subpopulation are displayed with and without hull in the center, with 5 mm scale bar. (b) Geographic distribution of these accessions. Number of countries sampled in each geographical region are indicated on top of the bars. (PDF 250 kb) [file 12915_2017_365_MOESM1_ESM.pdf]

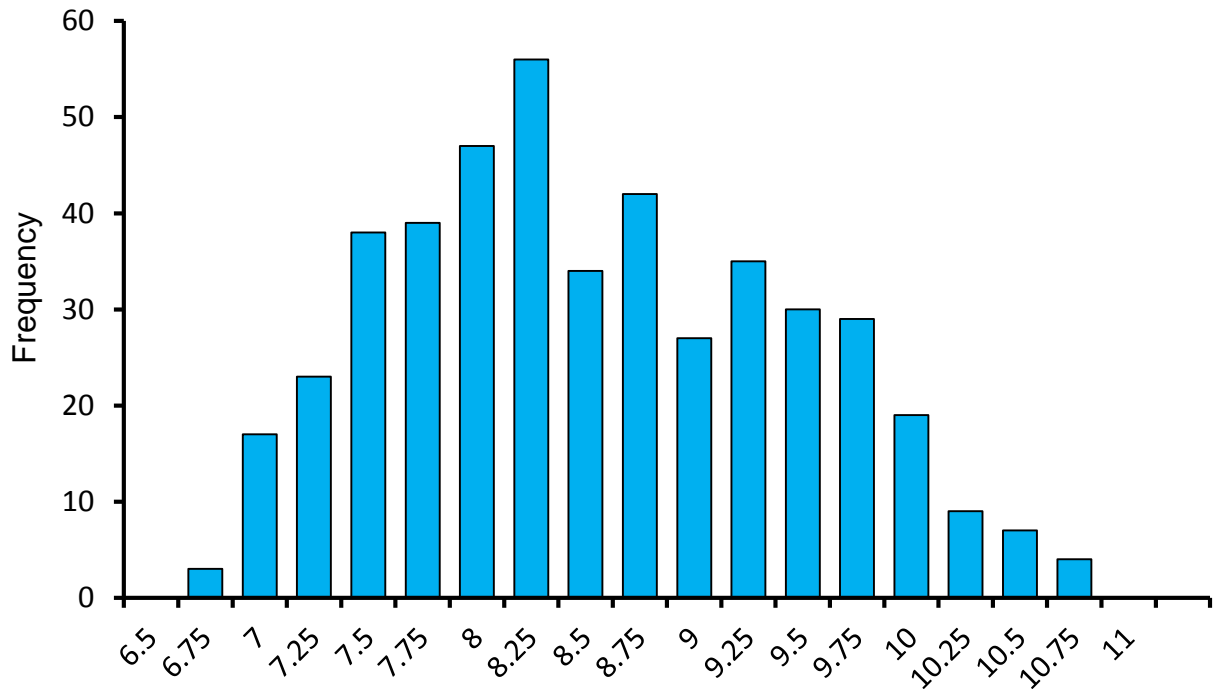

Supplement: Supplementary file 2 — Frequency distribution of grain length in the mini core collection (MCC population). (PDF 117 kb) [file 12915_2017_365_MOESM2_ESM.pdf]

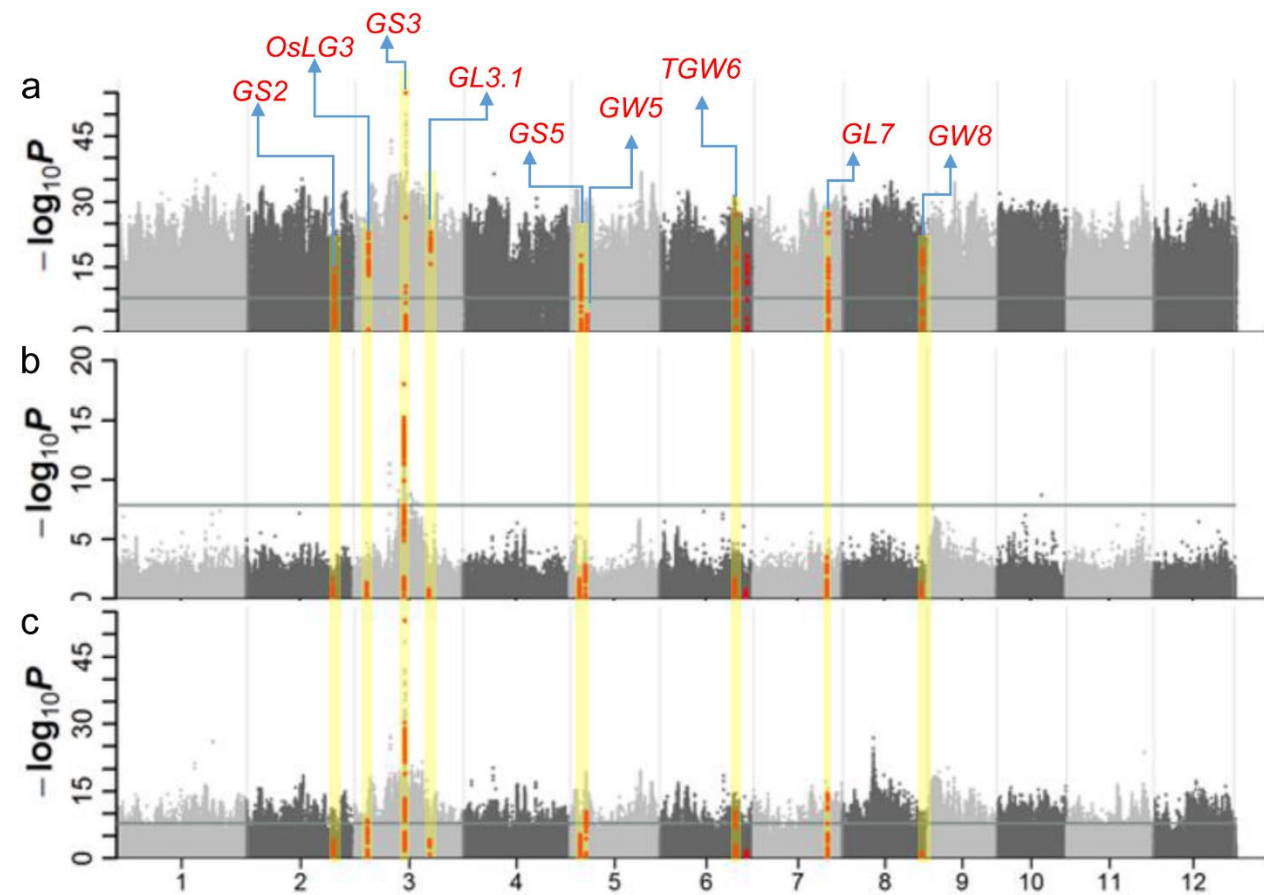

Supplement: Supplementary file 3 — Comparison of GWAS of grain length in the full population using LM model (a), CMLM model (b), and GLM (Q) model (c). In these Manhattan plots, for the significant loci identified, known loci are shown in red. Blue horizontal solid lines indicate the genome-wide significance threshold. (PDF 114 kb) [file 12915_2017_365_MOESM3_ESM.pdf]

**a**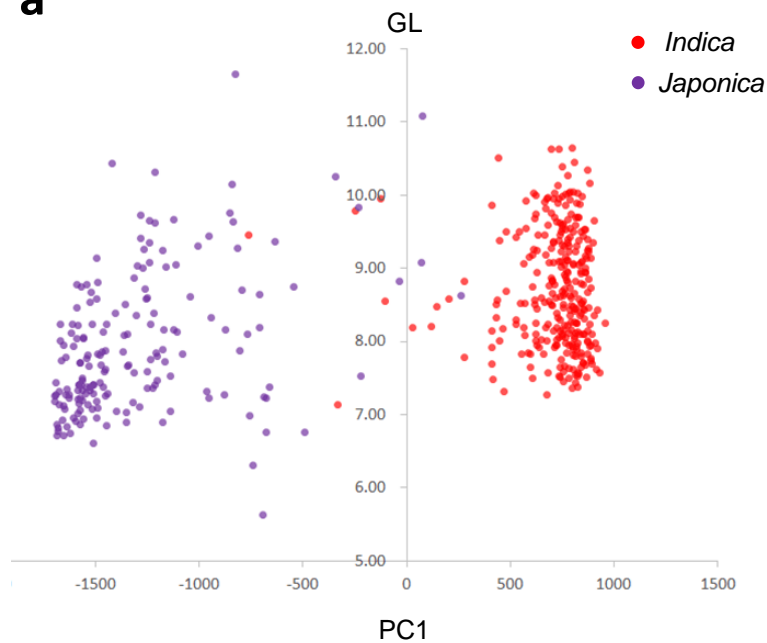**b**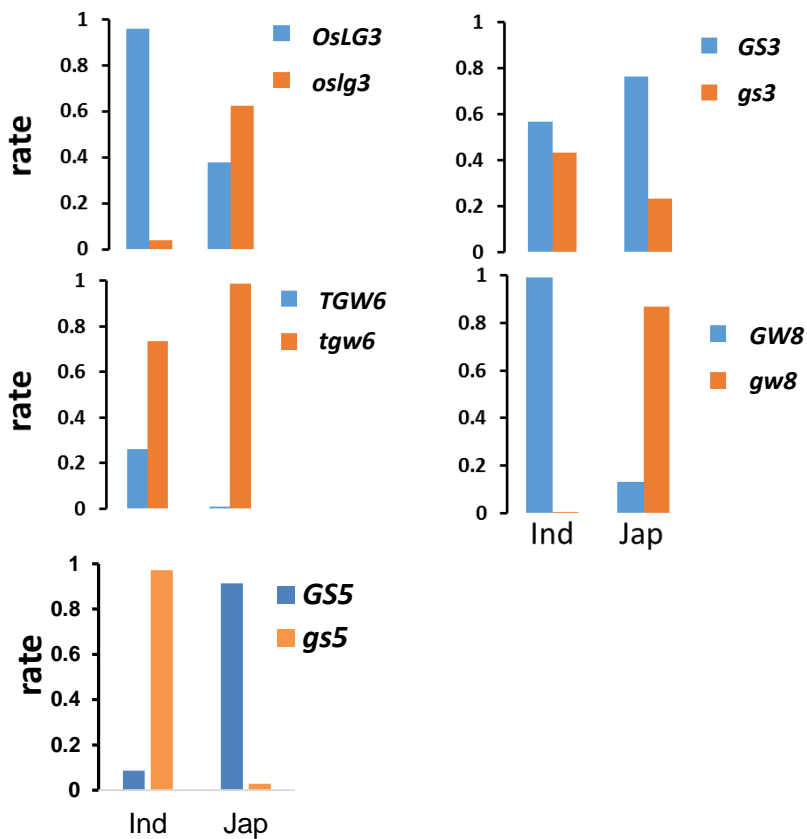

Supplement: Supplementary file 4 — Correlation between grain length and population structure in the natural population and frequency distribution between two genotypes in subpopulation about GS3, GS5, TGW6, GW8, and OsLG3. (a) Distribution of germplasms with different grain length according to PC structure. (b) Frequency distribution between two genotypes in subpopulation about GS3, GS5, TGW6, GW8, and OsLG3. The rate of each haplotype under two sub population divided by Q for these genes. Ind, indica; jap, japonica. (PDF 246 kb) [file 12915_2017_365_MOESM4_ESM.pdf]

## Slide 1
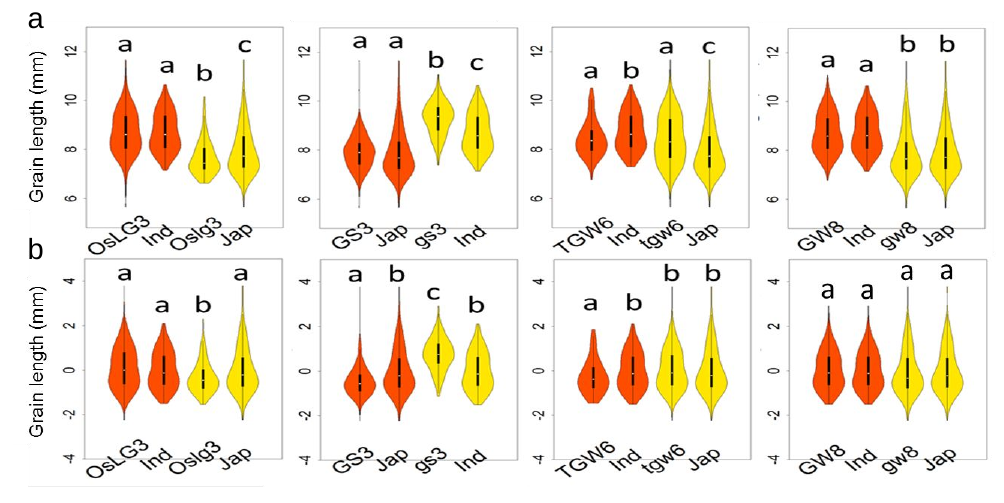

a
b

Supplement: Supplementary file 5 — Effect of Q structure (indica and japonica) for grain length. (a) Comparison of grain length between big-grain-haplotype and small-grain-haplotype when Q structure exists. (b) Comparison of grain length between big-grain-haplotype and small-grain-haplotype when the phenotype variation from Q structure was removed. Ind, indica; jap, japonica. Data are means ± SEM. Letters indicate a significant difference at P < 0.01 (n = 3) by the Student’s t-test. (PPTX 362 kb) [file 12915_2017_365_MOESM5_ESM.pptx]

Navie model

GLM(Q) model

MLM model

*OsLG3*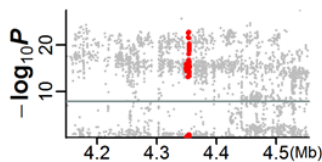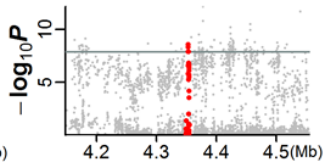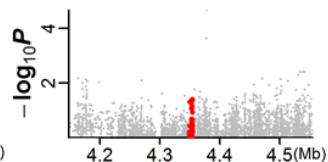*GS3*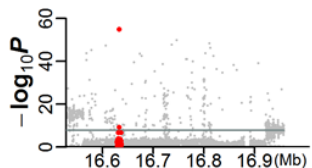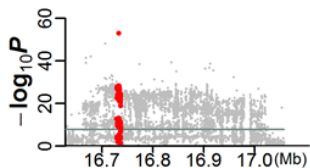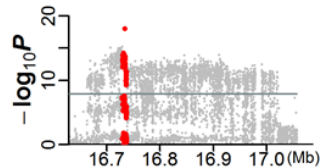*TGW6*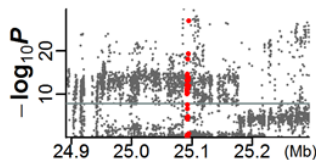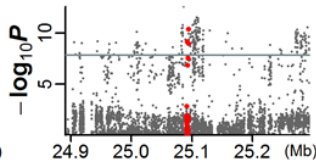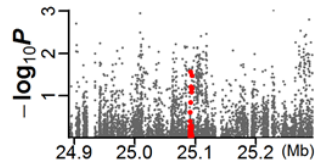*GW8*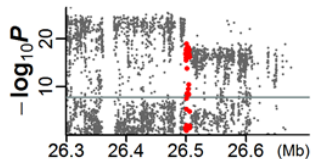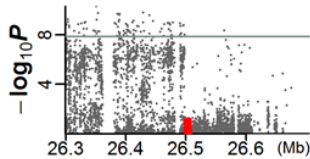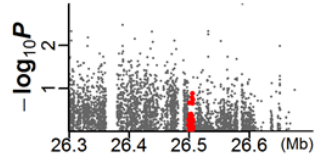

Supplement: Supplementary file 6 — Size of association regions about GS3, TGW6, GW8, and OsLG3. Manhattan plots in candidate region of three known genes and OsLG3 in different models. The red points indicate their SNPs within gene region, respectively. The horizontal full lines indicate the genome-wide significance threshold (0.05/n). (PDF 311 kb) [file 12915_2017_365_MOESM6_ESM.pdf]

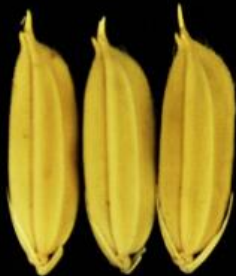

**SLG-1**

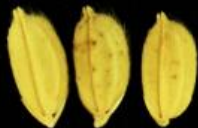

**Nipponbare**

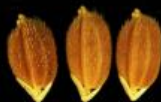

**chuanqi**

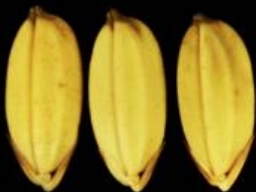

**Haobuka**

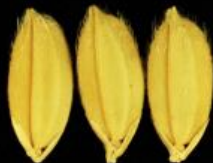

**IRAT109**

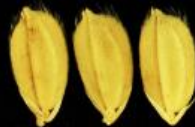

**YueFu**

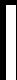

Supplement: Supplementary file 7 — Grains from six parents. Scale bar, 5 mm. (PDF 109 kb) [file 12915_2017_365_MOESM7_ESM.pdf]

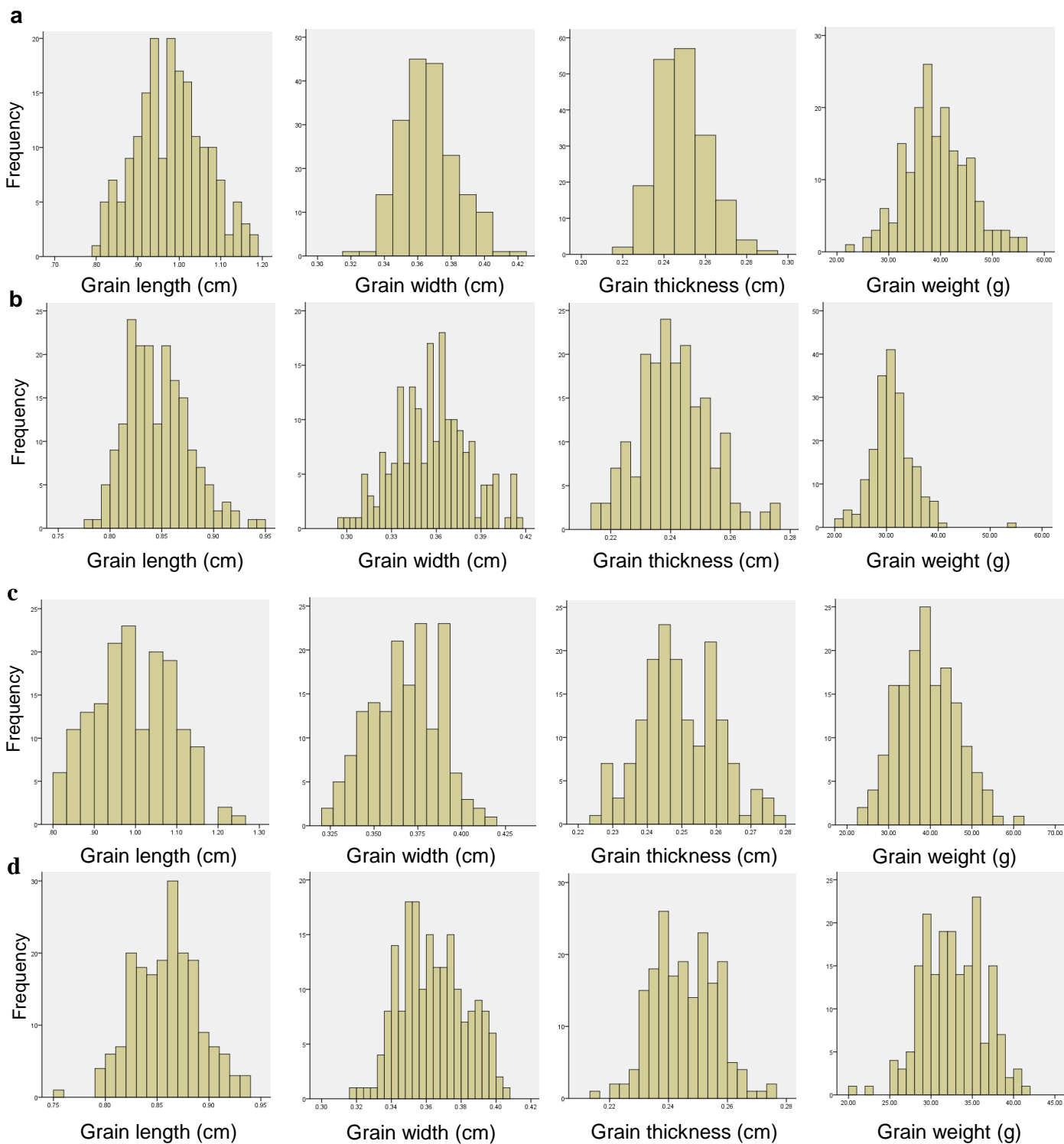

Supplement: Supplementary file 8 — Frequency distribution of variation of grain traits in biparental populations derived from NIP and SLG. (a–d) Phenotype variants of grain traits for populations 07DH010, 07DH011, 07DH013, and 07DH014, respectively. (PDF 103 kb) [file 12915_2017_365_MOESM8_ESM.pdf]

a

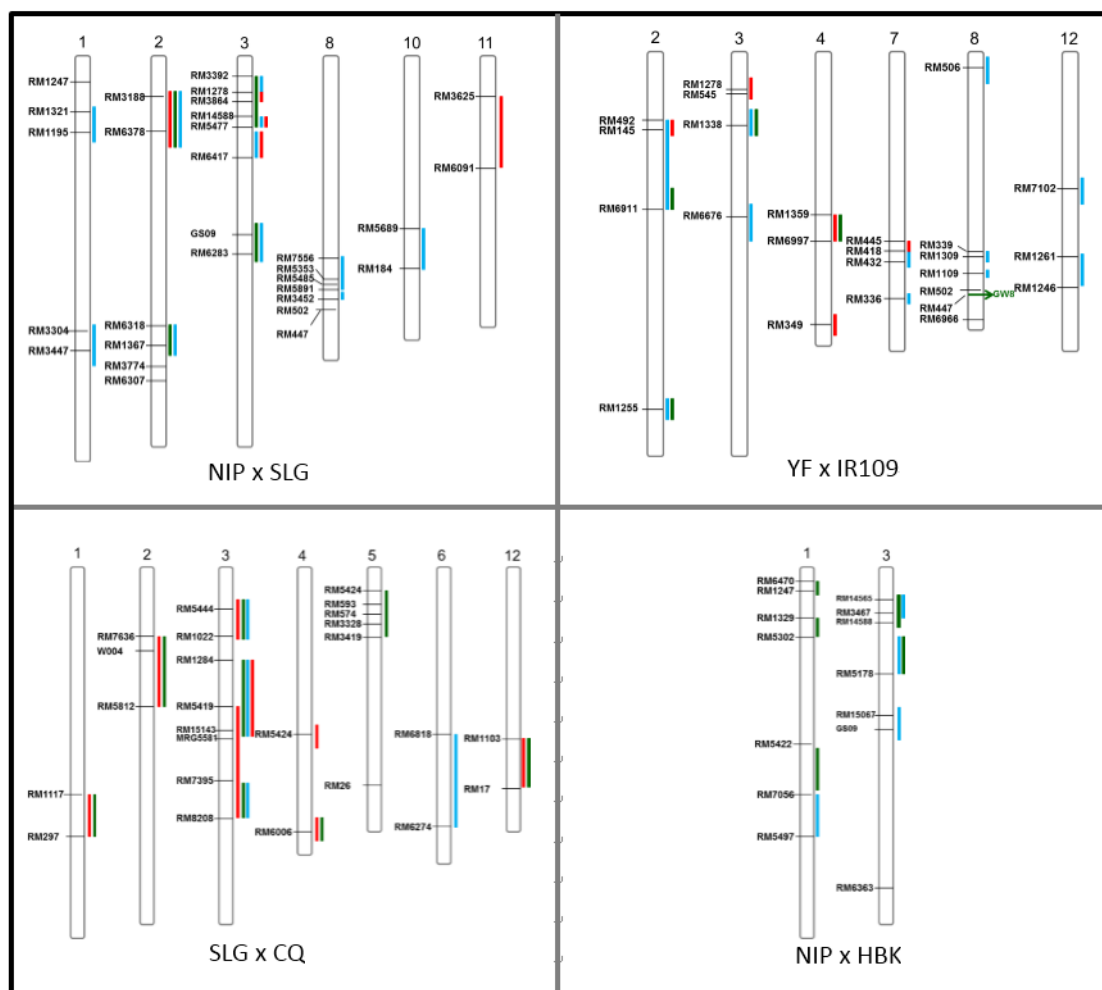

b

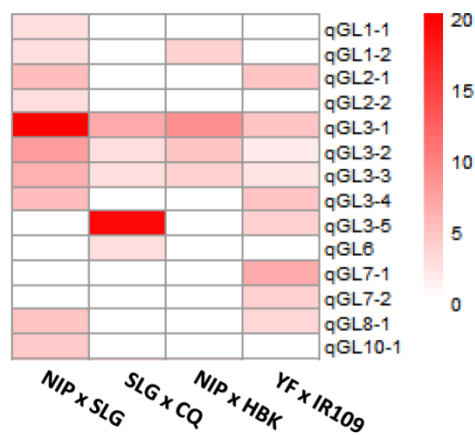

Supplement: Supplementary file 9 — QTL detected in the four crosses derived from six varieties. (a) Distribution of grain shape QTL and genes on genetic linkage map. Blue strip refers to grain length QTL, green to 1000-grain weight and grain width QTL, red to grain thickness QTL and arrows to cloned genes. (b) Heat map for effect of grain length QTL mapped by four linkage populations. Rows of the heat map correspond to the 14 QTL for grain length. NIP, Nipponbare; SLG, SLG-1; CQ, Chuanqi; YF, Yuefu; IR109, IRAT109; HBK, Haobuka. (PDF 224 kb) [file 12915_2017_365_MOESM9_ESM.pdf]

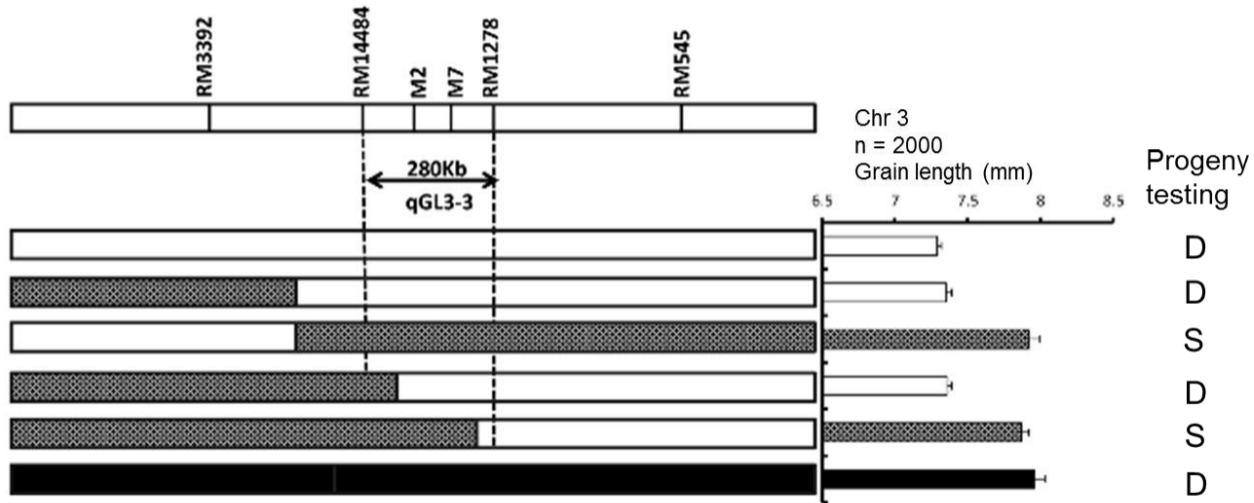

Supplement: Supplementary file 10 — Fine mapping of qGL3-3. White bars represent chromosomal segments for NIP homozygote, black for SLG homozygote, and grille for heterozygote, respectively. Progeny testing was used to confirm the genotypes at the qGL3-3 locus. S, segregation; D, desegregation. (PDF 78 kb) [file 12915_2017_365_MOESM10_ESM.pdf]

a

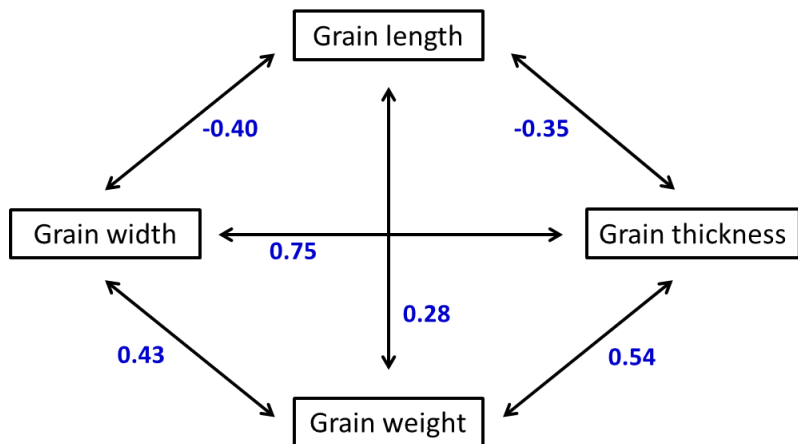

b

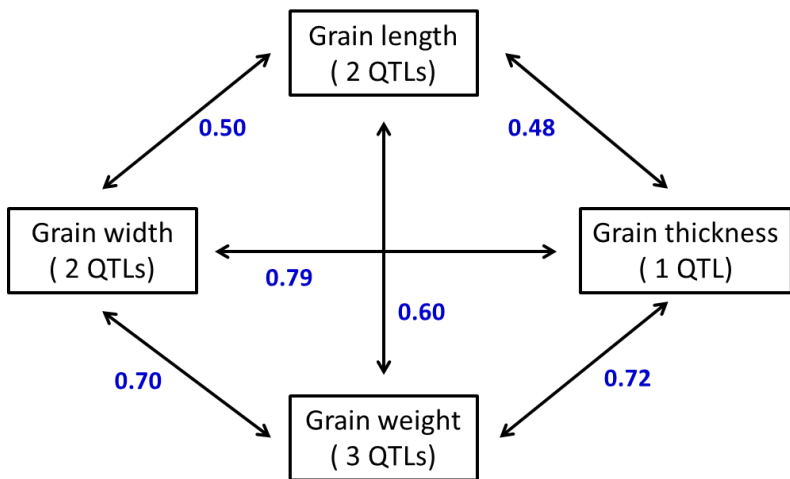

Supplement: Supplementary file 11 — Correlations between the grain traits in the MCC panel (a) and 07DH014 population (b). Number in blue, phenotypic correlation in r2 between traits. (PDF 124 kb) [file 12915_2017_365_MOESM11_ESM.pdf]

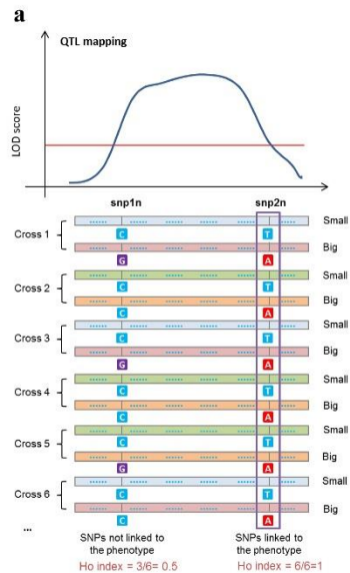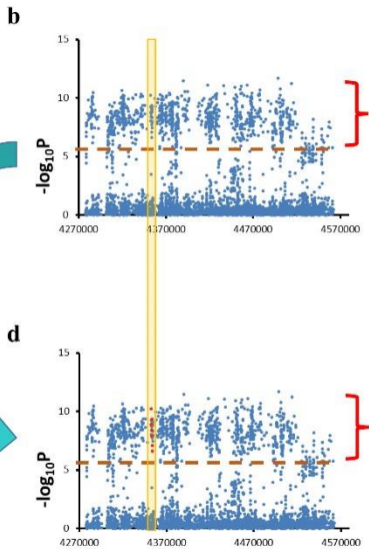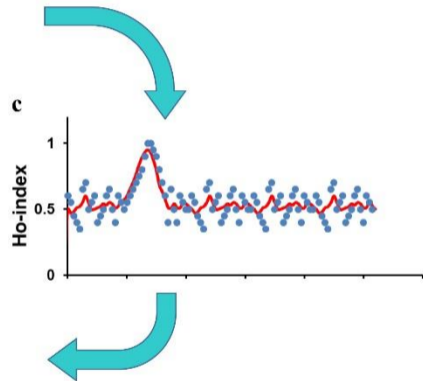

Supplement: Supplementary file 12 — Simplified scheme for application of Ho-LAMap to rice. (a) We cross diverse founder varieties (i.e., variety that is significantly different to the reference parent on grain traits) with reference parent (usually has small grain). The founder varieties are deep sequenced by the second-generation sequencing platforms, as in Additional file 30: Figure S30. In several crosses that have detected targeted QTL, the majority of SNPs between the QTL interval will segregate in a 1:1 founder varieties:reference parent ratio. However, the SNP responsible for the change of phenotype is the same in all founder parents, which can detect the targeted QTL. If we define the Ho (observed heterozygosity per locus) index as the ratio between the number of heterozygous crosses corresponding to each SNP locus and the total number of crosses which have detected targeted QTL, we expect this index would equal 1 near the causal SNP and 0.5 for the unlinked loci. (b, d) Candidate region association mapping. The brown horizontal dashed lines indicate the genome-wide significance threshold. The red points indicate significant loci within candidate gene. (c) Ho index plots for the target QTL. Red regression lines were obtained by averaging SNP indices from a sliding window analysis. (PDF 105 kb) [file 12915_2017_365_MOESM12_ESM.pdf]

## Slide 1
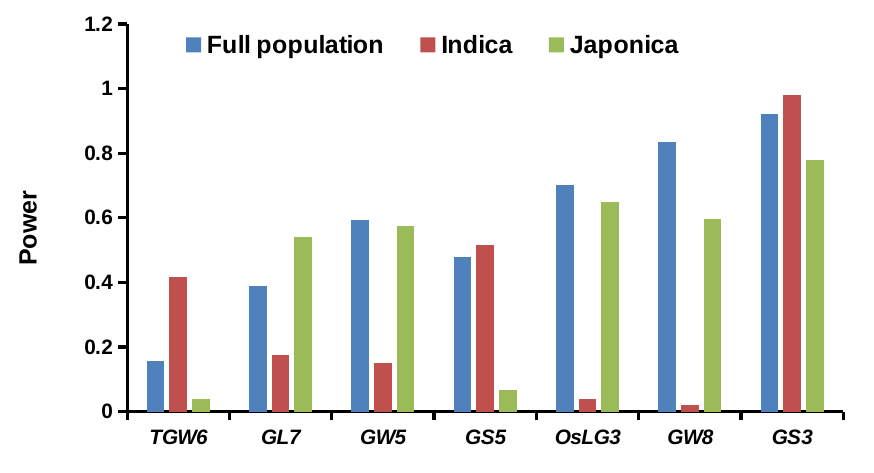

### Chart
| Category | Full population | Indica | Japonica |
|---|---|---|---|
| TGW6 | 0.155 | 0.416666666666667 | 0.04 |
| GL7 | 0.387351778656126 | 0.173913043478261 | 0.540909090909091 |
| GW5 | 0.592 | 0.1504 | 0.576 |
| GS5 | 0.478260869565217 | 0.515454545454545 | 0.0681818181818182 |
| OsLG3 | 0.7024 | 0.04 | 0.6496 |
| GW8 | 0.836 | 0.02 | 0.596 |
| GS3 | 0.920138888888889 | 0.979166666666667 | 0.778985507246377 |Power

Supplement: Supplementary file 13 — Simulation reveals effectiveness of Ho-LAMap in different subgroups for several known genes (such as GW8, TGW6, etc.) about grain size when using Ho-LAMap. The subgroups contain indica and japonica population. (PPTX 671 kb) [file 12915_2017_365_MOESM13_ESM.pptx]

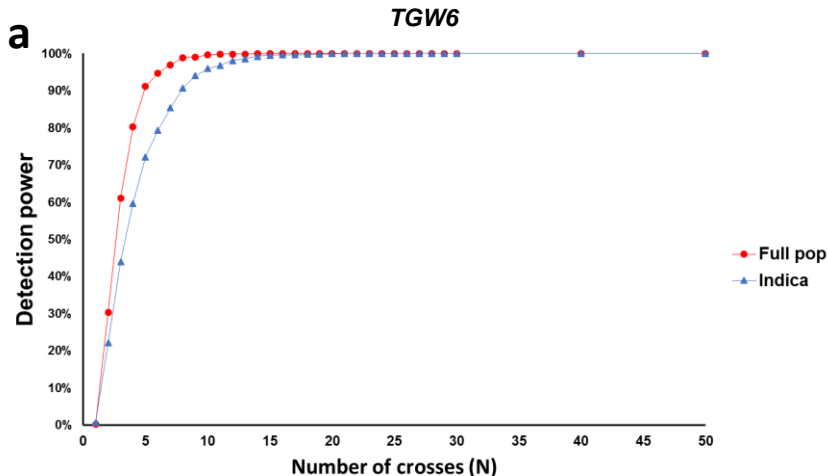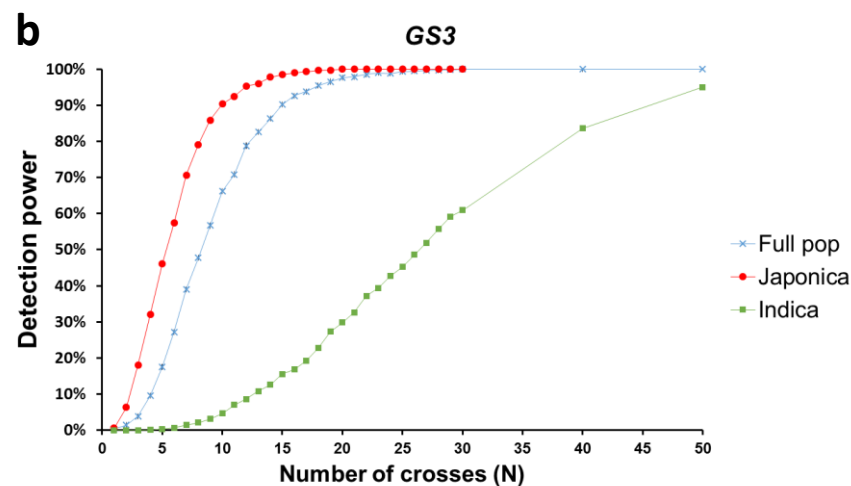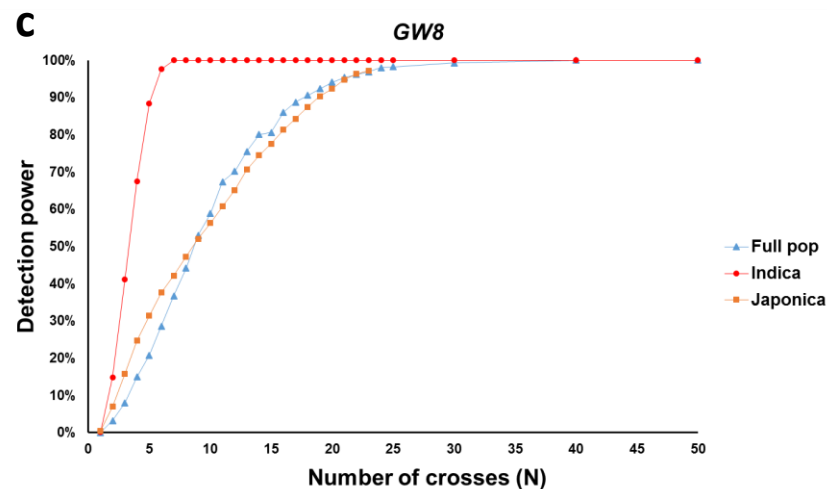

Supplement: Supplementary file 14 — Simulation reveals the cross number needed for three known genes for grain size when using Ho-LAMap. Simulation of crosses number also used significant SNPs of some of these well characterized genes (i.e., TGW6 (a), GS3 (b), and GW8 (c)). The x-axis value indicates cross number. Full pop, the full population; indica and japonica, the indica and japonica subgroup. We evaluated the power as the probability of detecting the target gene successfully in 1000 replications of the simulation. (PDF 227 kb) [file 12915_2017_365_MOESM14_ESM.pdf]

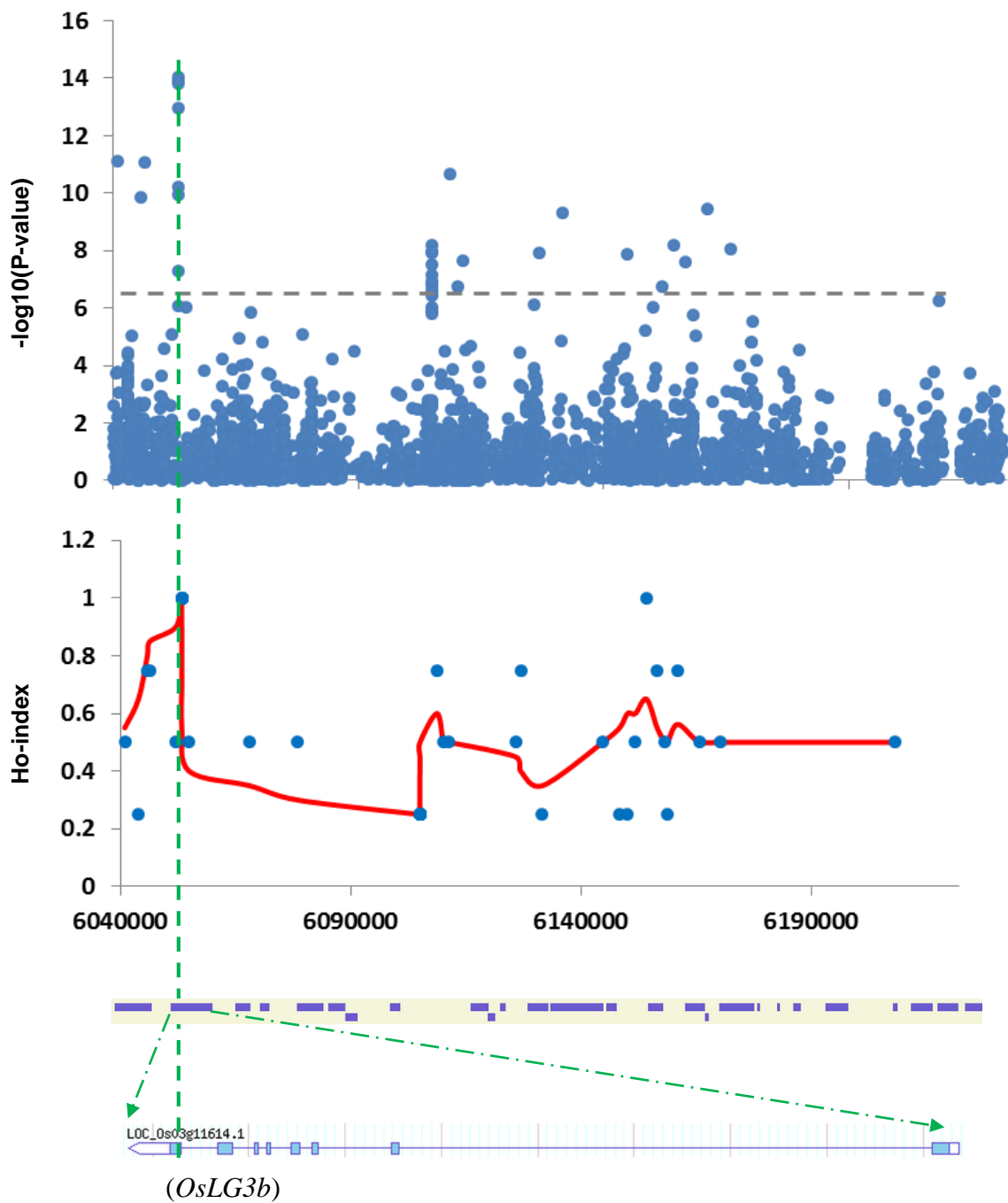

Supplement: Supplementary file 15 — Identification of the causal SNPs of QTL qGL3-2 using Ho-LAMap. We also used Ho-LAMap to clone OsLG3b from qGL3-2, a new gene for grain length, which encodes a MADS-box transcription factor. The top is Manhattan plot for candidate region association mapping for QTL region of qGL3-2; the middle correspond to Ho index plots for QTL region of qGL3-2. The bottom correspond to candidate gene (OsLG3b), the green dashed lines label the region for significant signal. (PDF 82 kb) [file 12915_2017_365_MOESM15_ESM.pdf]

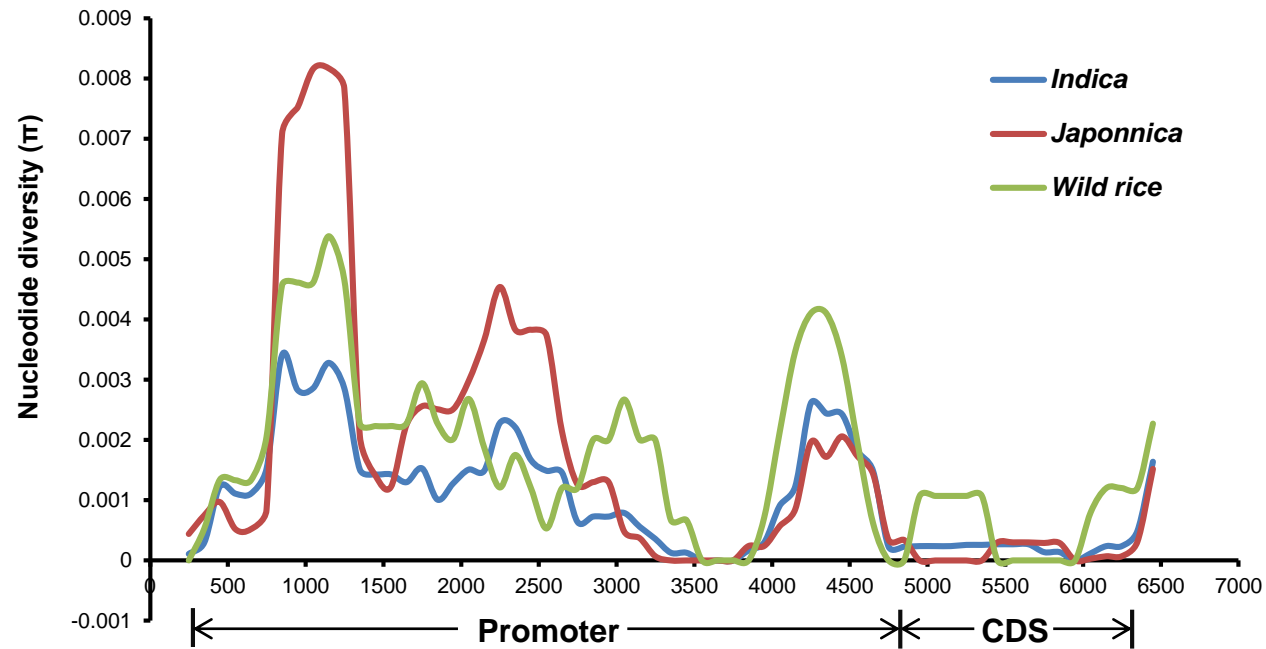

Supplement: Supplementary file 16 — Nucleotide diversity analysis for the promoter region and CDS of OsLG3. (PDF 59 kb) [file 12915_2017_365_MOESM16_ESM.pdf]

■ NIP ■ NIL(SLG)

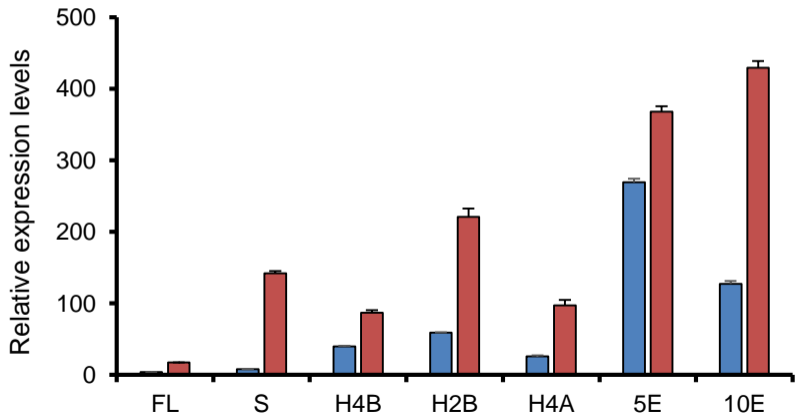

Supplement: Supplementary file 17 — OsLG3 expression levels in organs from NIP and NIL(SLG) plants. FL, flag leaf at the heading date; S, stem; H4B and H2B, hulls at 4 and 2 days before heading; H4A, hull at 4 days after heading; 5E and 10E, endosperm at 5 and 10 days after fertilization; n = 3. Data are given as mean ± SEM. (PDF 84 kb) [file 12915_2017_365_MOESM17_ESM.pdf]

**a**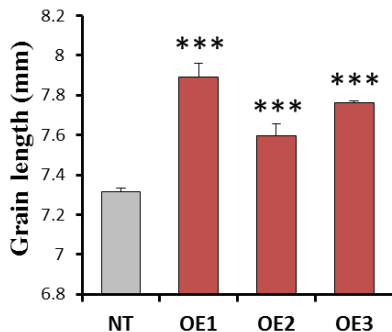**b**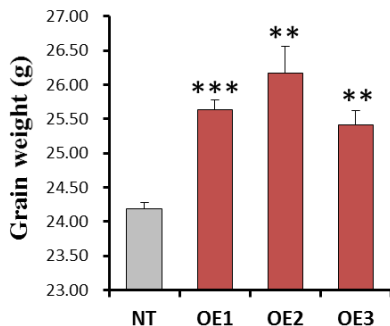**c**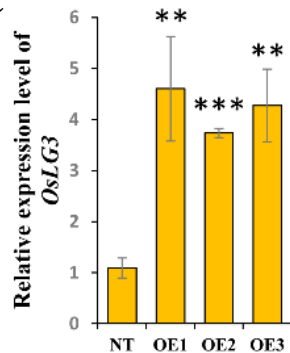**d**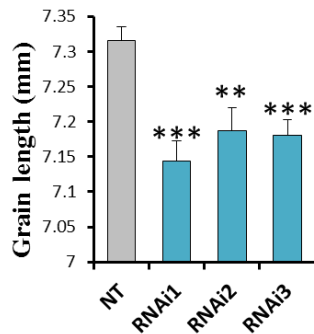**e**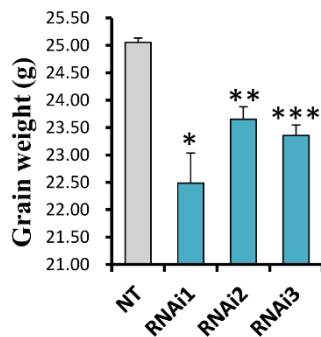**f**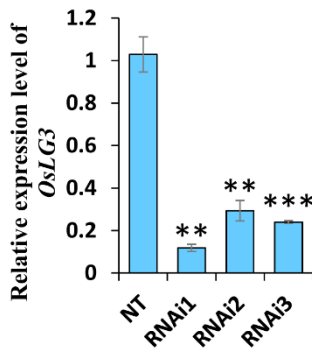

Supplement: Supplementary file 18 — Phenotypes of grain in three OsLG3-overexpressing lines and three RNAi-OsLG3 lines. (a, d) Grain length of the transgenic and control lines (n = 30). (b, e) 1000-grain weight in the transgenic and control lines (n = 30). (c, f) Relative expression levels of OsLG3 in young panicles of the transgenic and control lines were detected by qPCR, with data normalized to OsActin1 levels (n = 3). All data in c, f–h, and j–l are presented as means ± SEM. **P < 0.01, Student’s t test. (PDF 77 kb) [file 12915_2017_365_MOESM18_ESM.pdf]

**a**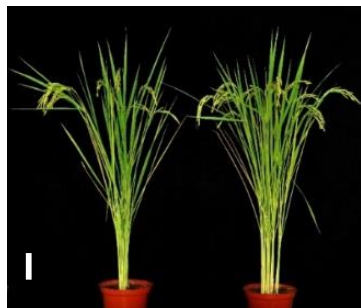

NIP

NIL(SLG)

**b**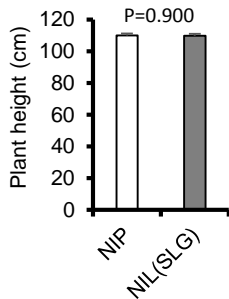**c**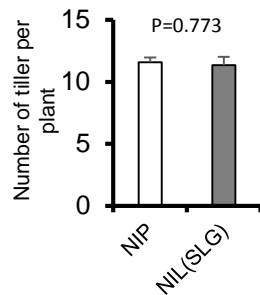**d**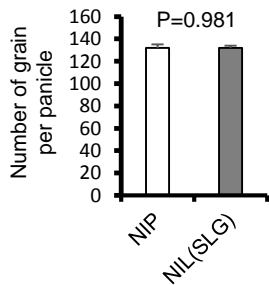**e**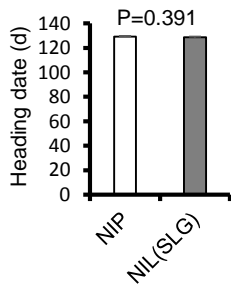

Supplement: Supplementary file 19 — A field trial of NIP and NIL(SLG) plants. (a) The morphology of the NIL plants. Scale bar, 10 cm. (b) Plant height. (c) Tiller number. (d) Number of grains per panicle. (e) Heading date. All phenotypic data in b–e were measured from plants grown with 20 × 20 cm spacing in paddies under normal cultivation conditions. Data represent mean ± SEM. (n = 30). Student’s t tests were used to generate P values. (PDF 111 kb) [file 12915_2017_365_MOESM19_ESM.pdf]

**a****NIP****NIL(SLG)**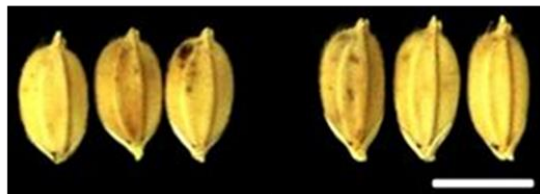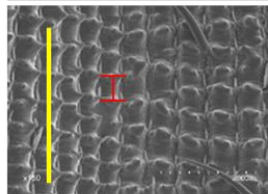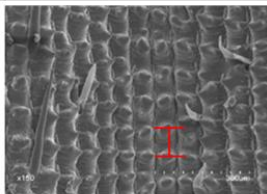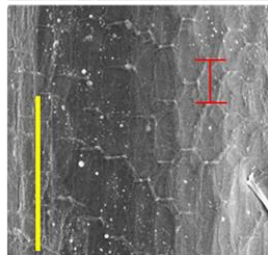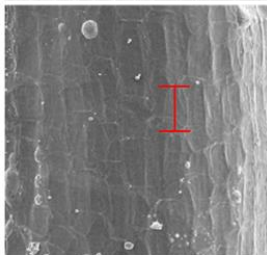**b**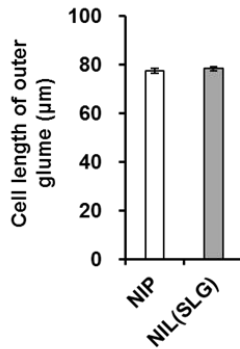**c**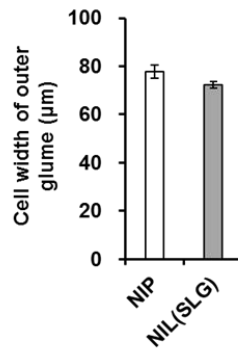**d**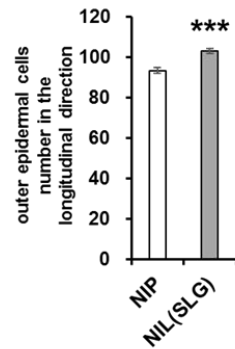**e**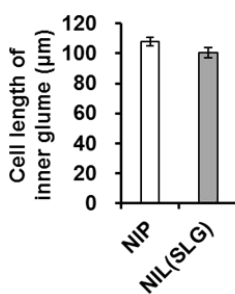**f**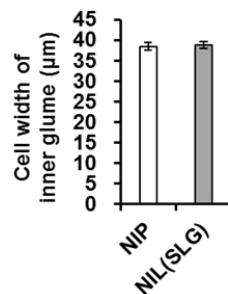**g**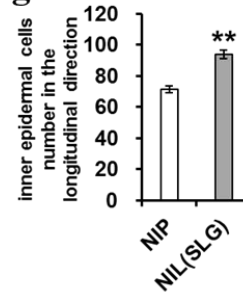

Supplement: Supplementary file 20 — OsLG3 regulates grain length by changing cell division patterns. (a) The grains of NIP and NIL(SLG) plants (top) and scanning electron microscope images of the outer glume (middle) and inner epidermal cells of the lemma (bottom) before anthesis. Scale bars, 5 mm (white; top) and 300 μm (yellow; middle and bottom). (b) Cell length of outer epidermal cells in the longitudinal direction in a (n = 12). (c) Cell width of outer epidermal cells in the longitudinal direction in (a) (n = 12). (d) Total cell number of outer epidermal cells in the longitudinal direction. (e) Cell length of inner epidermal cells in the longitudinal direction in (a) (n = 12). (f) Cell width of inner epidermal cells in the longitudinal direction in (a) (n = 12). (g) Total cell number of inner epidermal cells in the longitudinal direction. All data represent means ± SEM. *P < 0.05, **P < 0.01, Student’s t test. (PDF 134 kb) [file 12915_2017_365_MOESM20_ESM.pdf]

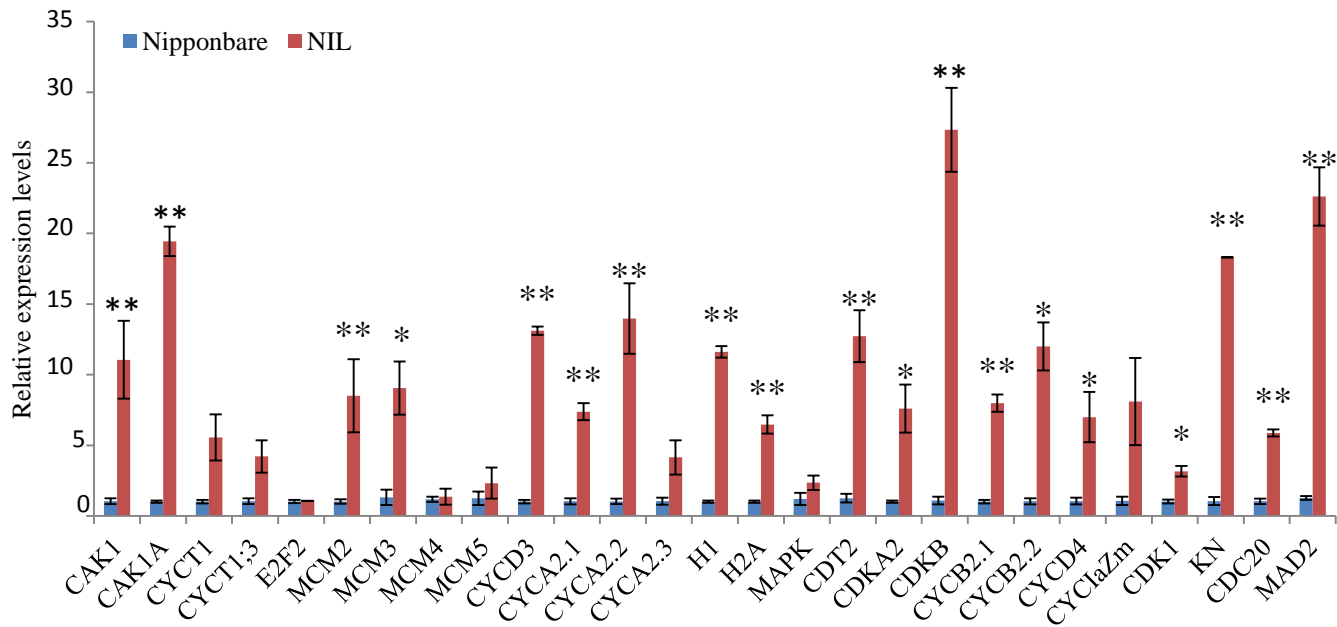

Supplement: Supplementary file 21 — The effect of OsLG3 on the expression of genes involved in cell cycle. The expression analysis was conducted using 7-cm long young panicles. OsActin1 was used as the control and the values of expression levels in Nipponbare were set to 1 (n = 3). Data are given as mean ± SEM. Student’s t-test was used to generate the P values; * P < 0.05, ** P < 0.01, respectively. (PDF 135 kb) [file 12915_2017_365_MOESM21_ESM.pdf]

## Slide 1
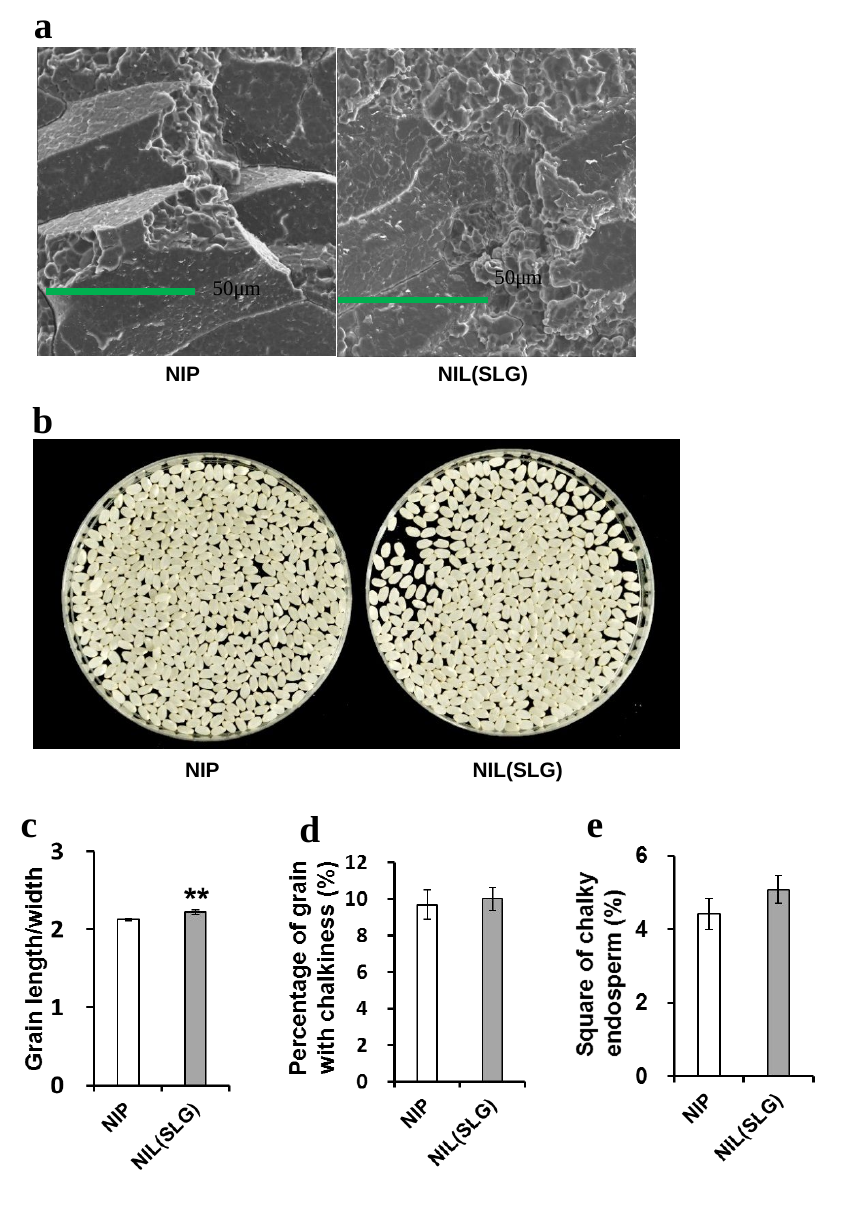

a
50μm
50μm
NIP
NIL(SLG)
b
NIP
NIL(SLG)
e
c
d

Supplement: Supplementary file 22 — OsLG3 does not affect grain quality. (a) Scanning electron microscopy images are transverse sections of starch granule of NIP and NIL(SLG). Scale bars, 50 μm (green line). (b) Comparison of brown grains NIP and NIL(SLG). Scale bar, 2 cm. (c) The ratio of grain length to width (n = 30). (d) Percentage of grain with chalkiness (%) (n = 6). (e) Square of chalky endosperm (%) (n = 6). Data are given as means ± SEM. Student’s t-test was used to generate the P values; * P < 0.05, ** P < 0.01, respectively. (PPTX 405 kb) [file 12915_2017_365_MOESM22_ESM.pptx]

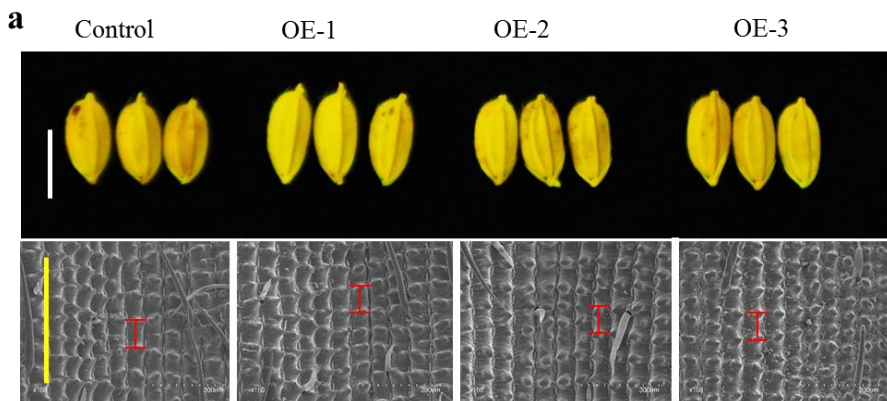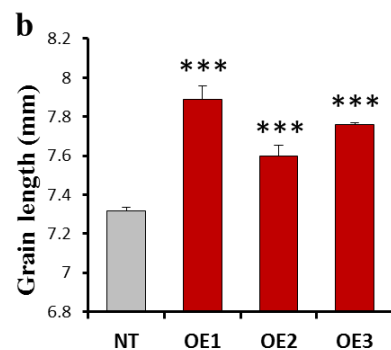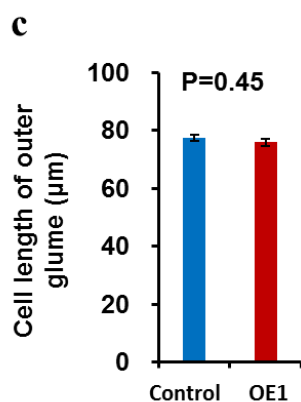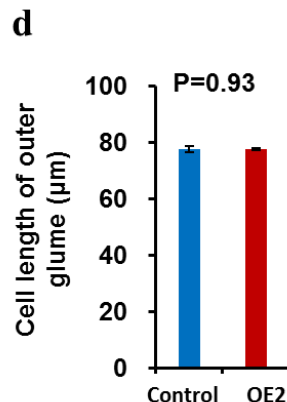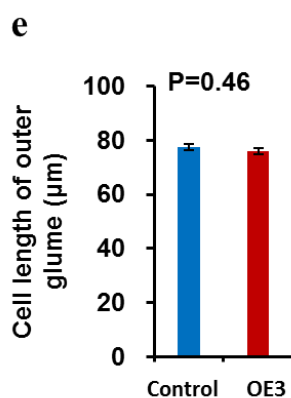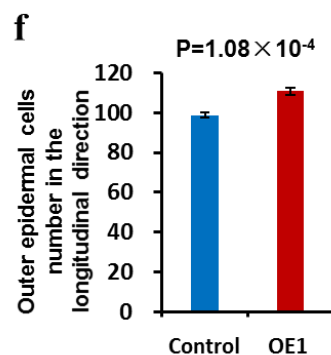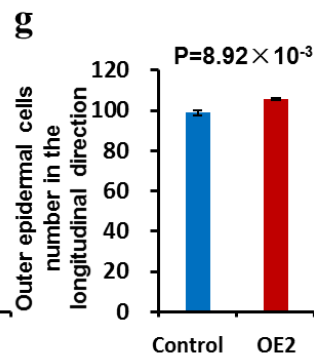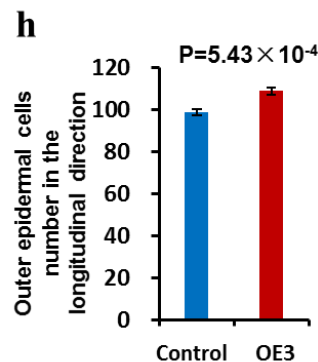

Supplement: Supplementary file 23 — Overexpression of OsLG3 in NIP has large effect on grain length and cell number. (a) The mature rice grain images of NIP (left) and over-expression lines (right), OE-3, OE-4, and OE-5, respectively. The scanning electron microscope images are the outer glume epidermal cells of the lemma from the spikelet hulls of NIP (left) and over-expression plant (right) before anthesis, respectively. Scale bars, 5.0 mm (white line) and 300 μm (yellow line), respectively. (b) Grain length. (c–e) Cell length of outer epidermal cells in the longitudinal direction in a (n = 12). (f–h) Total cell number of outer epidermal cells in the longitudinal direction (n = 12). All phenotypic data in b–h were measured from plants grown with 15 × 20 cm spacing in paddies under normal cultivation conditions. All data represent means ± SEM. *P < 0.05, **P < 0.01, Student’s t test. (PDF 107 kb) [file 12915_2017_365_MOESM23_ESM.pdf]

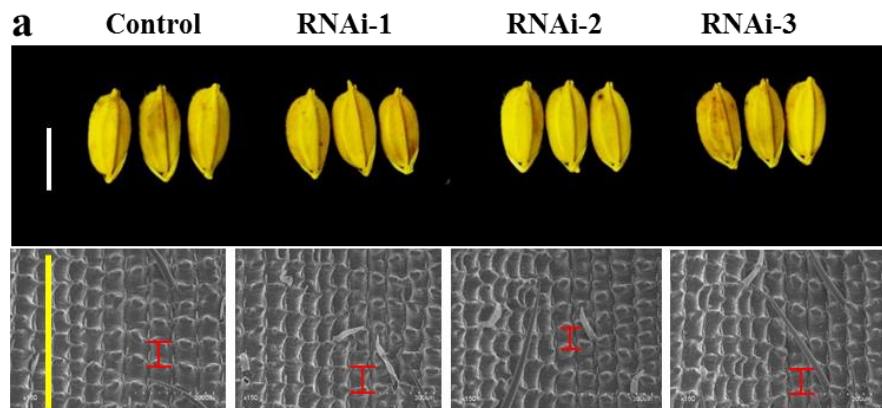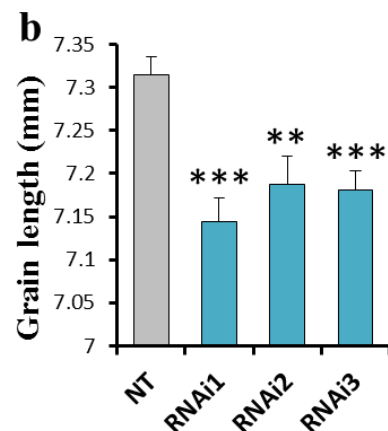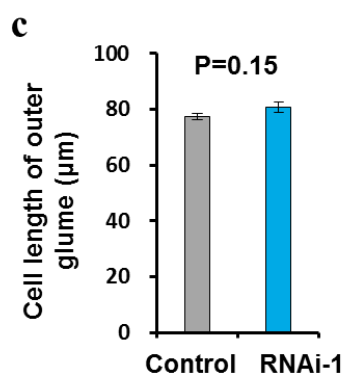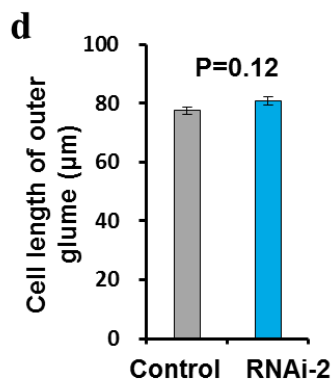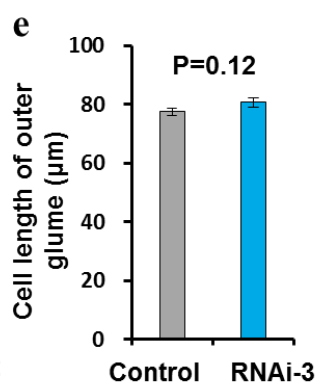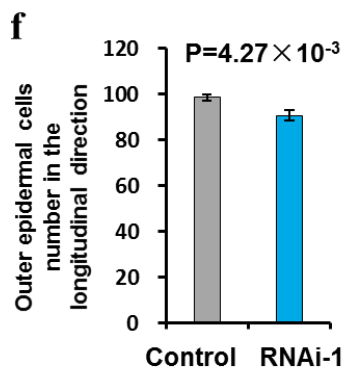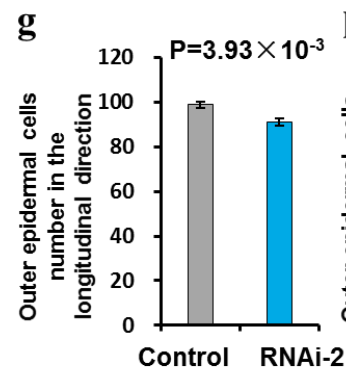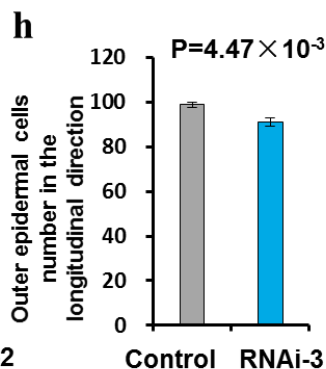

Supplement: Supplementary file 24 — Effect of RNAi-OsLG3 in NIP on grain length. RNAi-OsLG3 in NIP has large effect on grain length. (a) The mature rice grain images are control (left) and RNAi-1, RNAi-2, and RNAi-3, respectively (right). The scanning electron microscope images are the outer glume epidermal cells of the lemma from the spikelet hulls of NIP (left) and RNAi-OsLG3 plant (right) before anthesis, respectively. Scale bars, 5.0 mm (white line) and 300 μm (yellow line), respectively. (b) Grain length. (c–e) Cell length of outer epidermal cells in the longitudinal direction in a (n = 12). (f–h) Total cell number of outer epidermal cells in the longitudinal direction (n = 12). All phenotypic data in b–h were measured from plants grown with 15 × 20 cm spacing in paddies under normal cultivation conditions. All data represent means ± SEM. *P < 0.05, **P < 0.01, Student’s t test. (PDF 89 kb) [file 12915_2017_365_MOESM24_ESM.pdf]

a

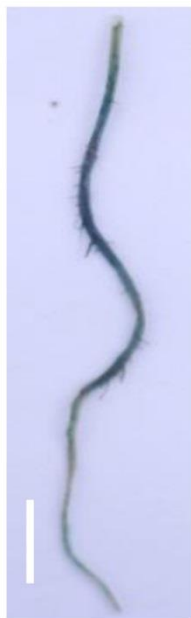

b

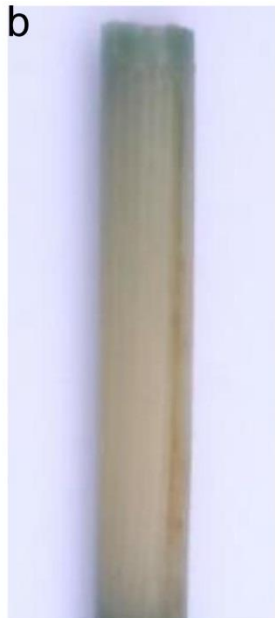

c

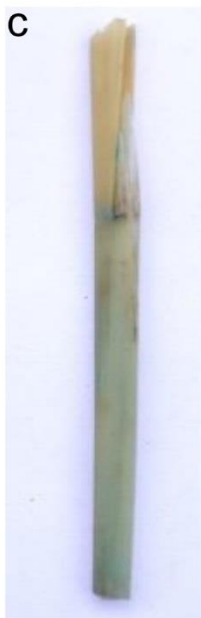

d

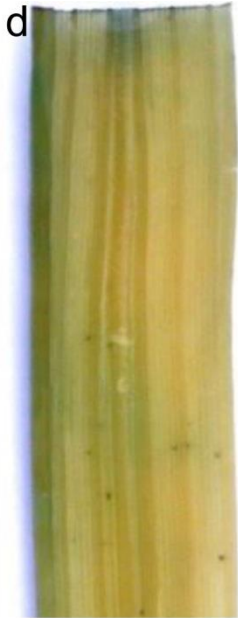

e

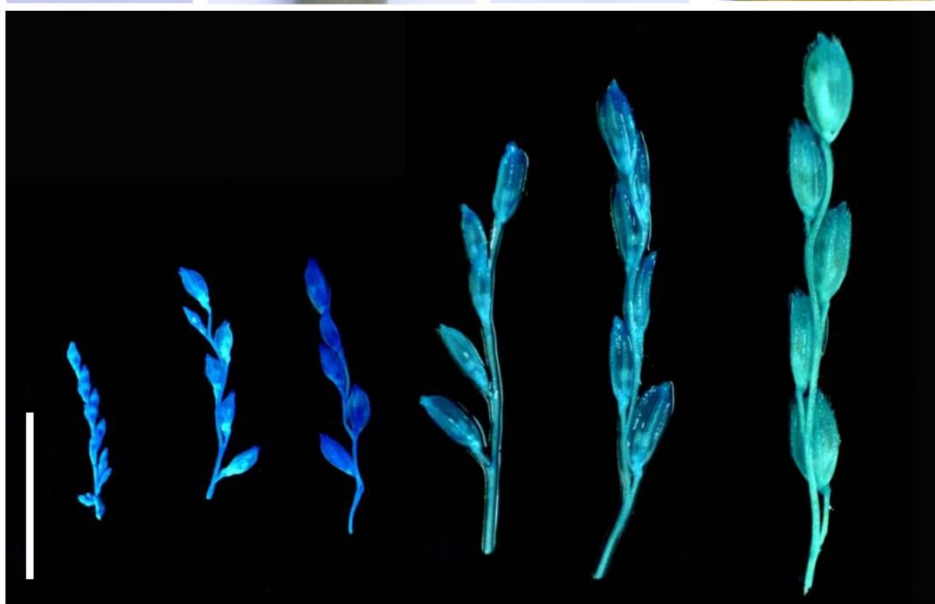

Supplement: Supplementary file 25 — OsLG3 expression activity was monitored by pOsLG3:GUS transgene expression. Histochemical analysis of GUS activity in root (a), stem (b), sheath (c), leaf (d), and the developing panicles (e). Scale bar, 1 cm. (PDF 90 kb) [file 12915_2017_365_MOESM25_ESM.pdf]

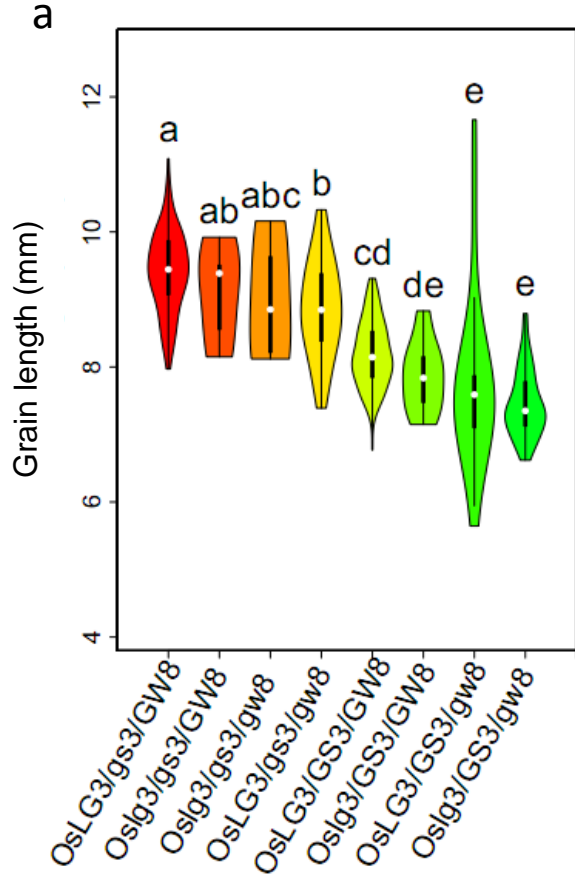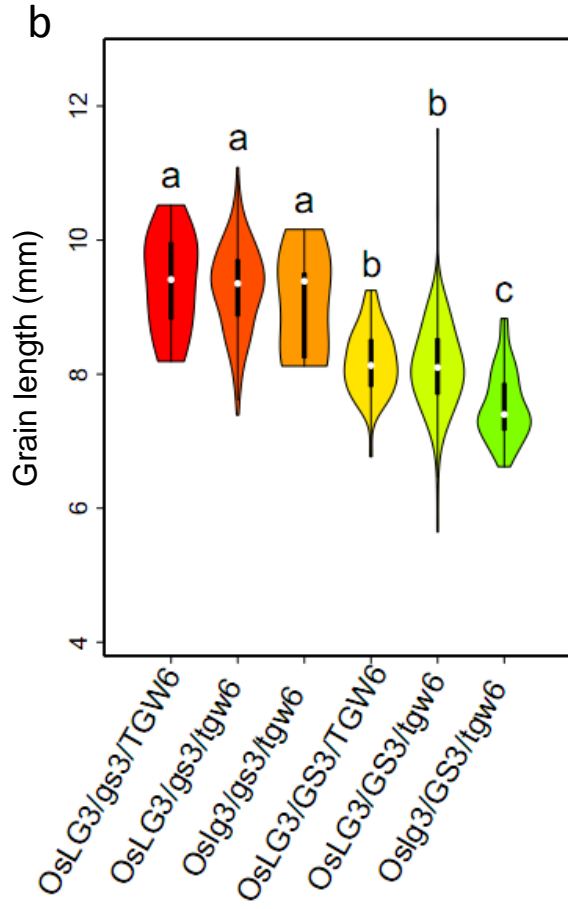

Supplement: Supplementary file 26 — Genetic interactions among OsLG3, GS3, GW8, and TGW6. (a) Varieties were categorized by allelic variations of OsLG3, GS3, and TGW6. (b) Varieties were categorized by allelic variations in the functional SNP of OsLG3, GS3, and GW8. Grain length was measured by Vernier calipers, and the violin map was constructed in R. Multiple comparisons were done by Tukey’ HSD in R. Landraces and raw data were listed in Additional file 32: Table S1. (PDF 143 kb) [file 12915_2017_365_MOESM26_ESM.pdf]

**a**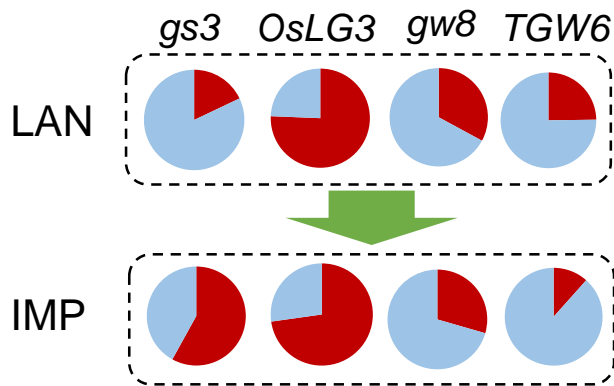**b**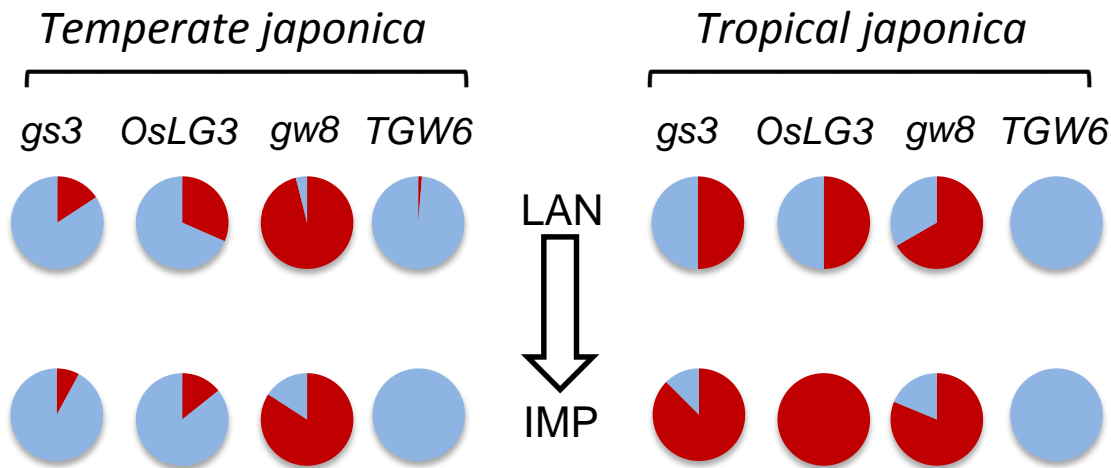

Supplement: Supplementary file 27 — The spectrum of allele frequencies between landrace and improved varieties at the causal polymorphisms of GS3, OsLG3, GW8, and TGW6 during modern breeding in full population (a) and japonica subgroup (b). LAN, landrace; IMP, improved variety. (PDF 199 kb) [file 12915_2017_365_MOESM27_ESM.pdf]

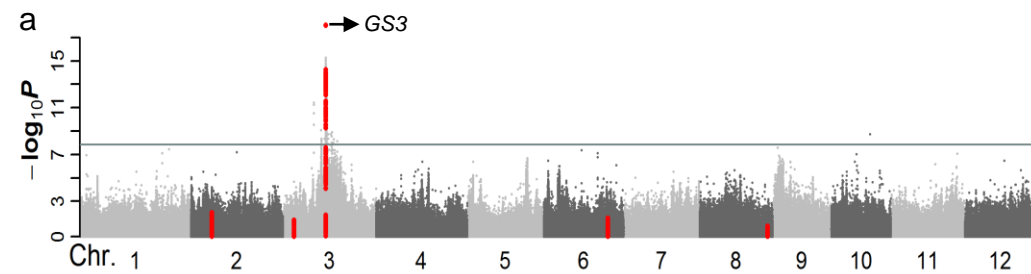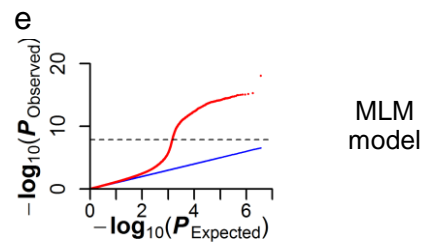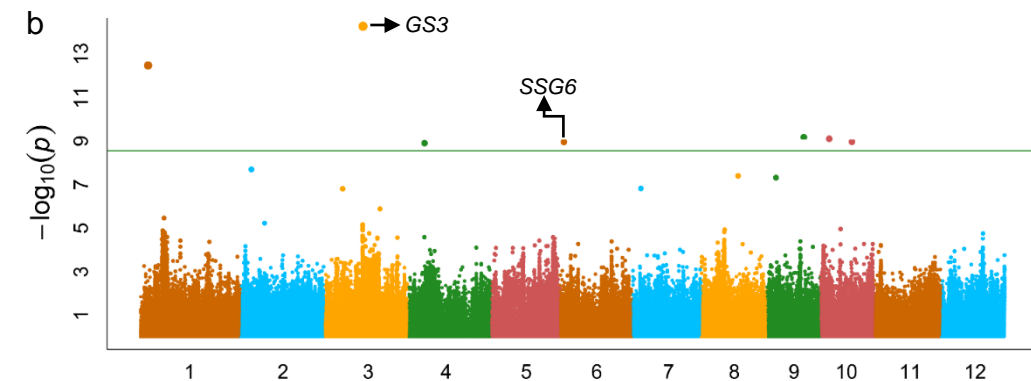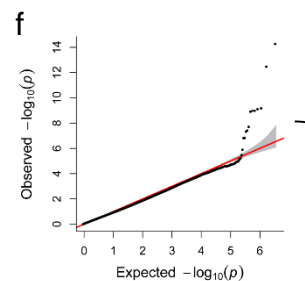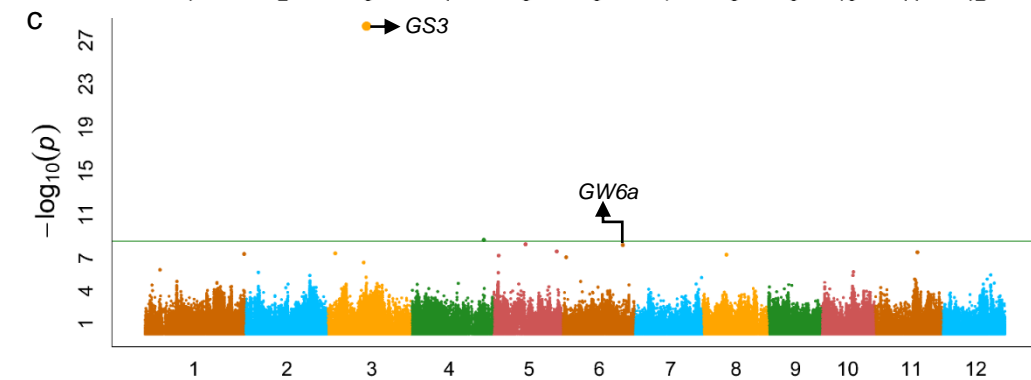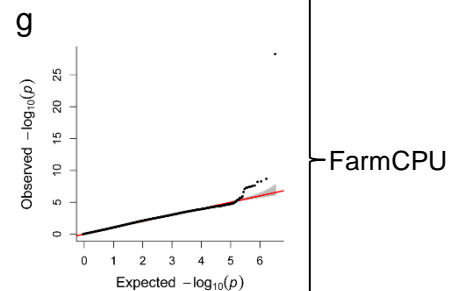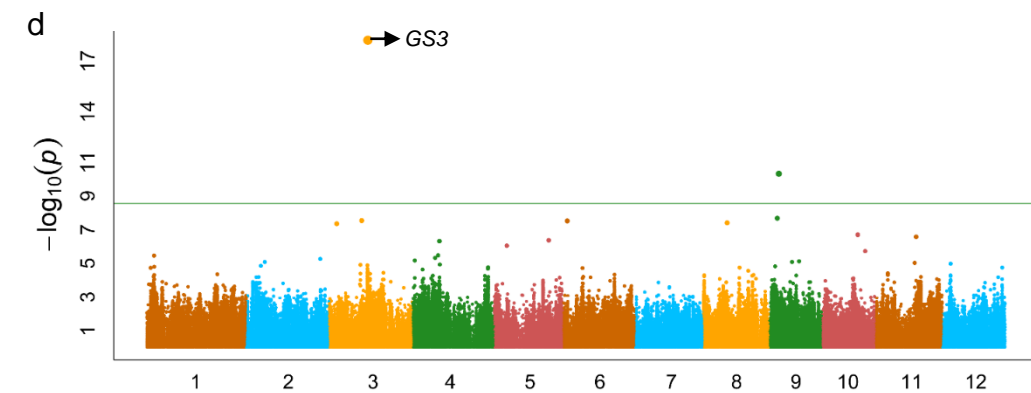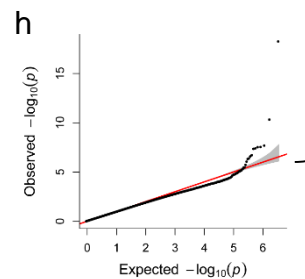

Supplement: Supplementary file 28 — Comparison of GWAS of grain length in the full population using MLM (a), FarmCPU included the first three PCs (b), two PCs (c), and one PC (d) as covariates. In these Manhattan plots, for the significant loci identified, five SNPs within a 200-kb range of known genes are marked as significant loci re-identified. Green horizontal solid lines in (b), (c), and (d) indicate the Bonferroni-corrected significant threshold (0.01/n). However, the genome-wide significance threshold of all methods in this study adopts the Bonferroni-corrected threshold with 0.05 (0.05/n) (see Additional file 38: Table S15). Quantile-quantile plots of these models are shown in e, f, g, and h, respectively. (PDF 261 kb) [file 12915_2017_365_MOESM28_ESM.pdf]

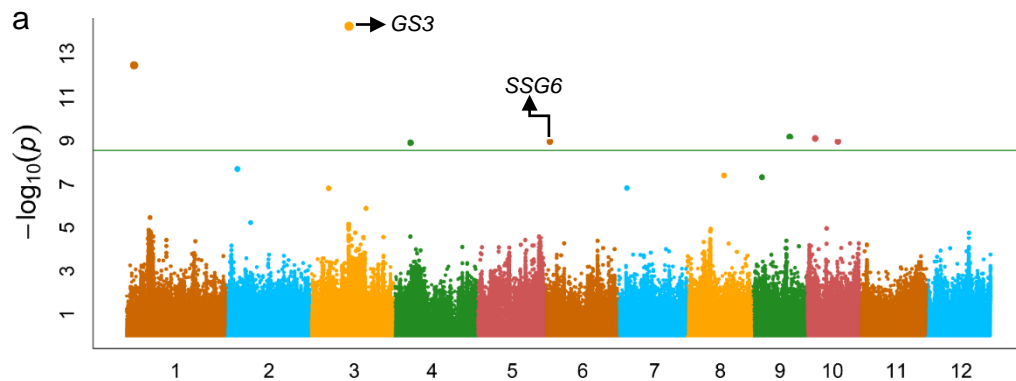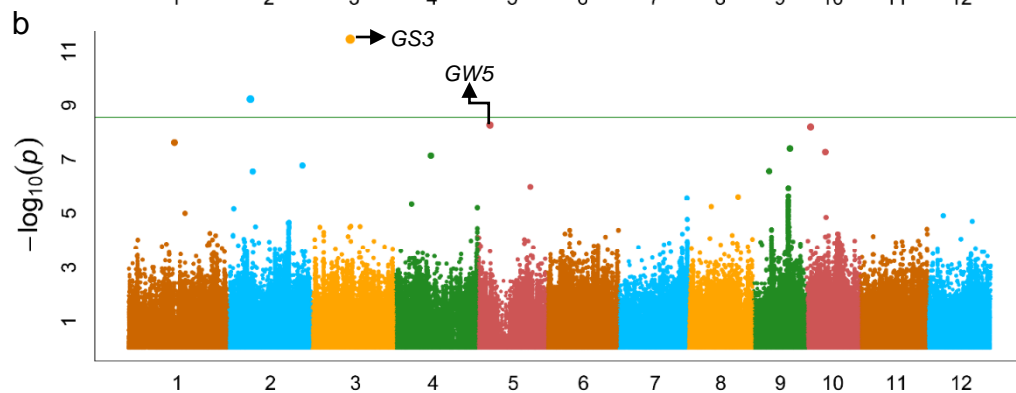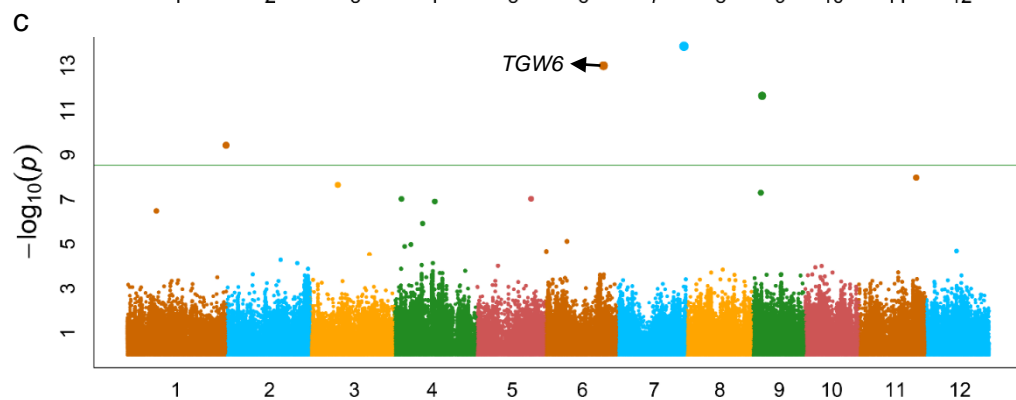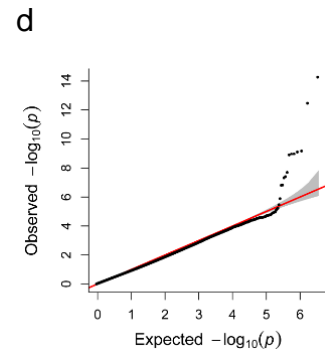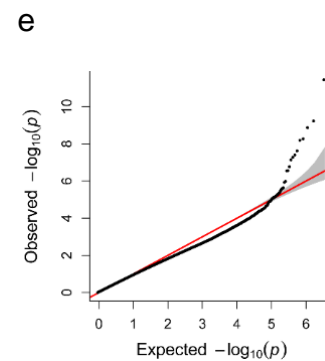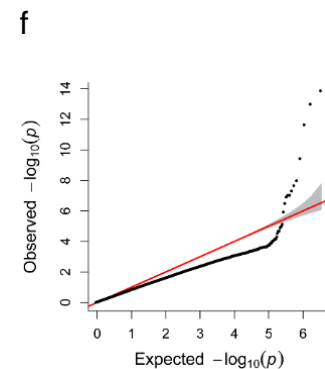

Supplement: Supplementary file 29 — Comparison of GWAS of grain length in the full population (a), indica (b), and japonica (c) subpopulations using FarmCPU included the first three PCs as covariates. In these Manhattan plots, for the significant loci identified, five SNPs within a 200-kb range of known genes are marked as significant loci re-identified. Green horizontal solid lines in (a), (b), and (c) indicate the Bonferroni-corrected significant threshold (0.01/n). However, the genome-wide significance threshold of all methods in this study adopts the Bonferroni-corrected threshold with 0.05 (0.05/n) (see Additional file 38: Table S15). Quantile-quantile plots in these three populations are shown in d, e, and f, respectively. (PDF 251 kb) [file 12915_2017_365_MOESM29_ESM.pdf]

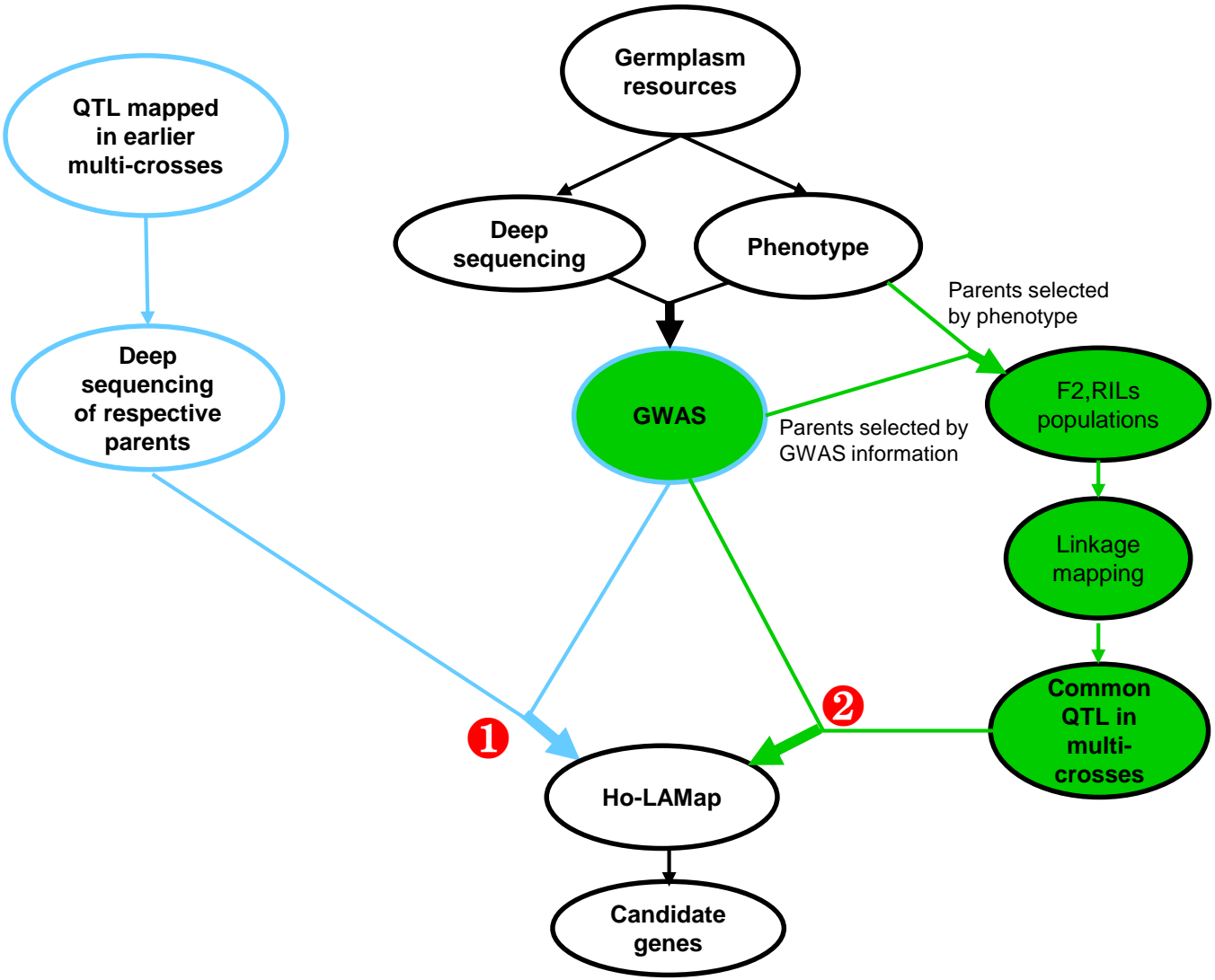

Supplement: Supplementary file 30 — Proposed strategy of gene discovery by Ho-LAMap. The number 1 and 2 in the red cycle indicate two pathways for gene discovery by Ho-LAMap. At the first way, a lot of QTL had been co-localized in many crosses by predecessors. Therefore, we re-sequenced parents of those crosses and isolated gene from the target QTL via Ho-LAMap. For the second way, we obtained the QTL region by GWAS and selected extreme materials to construct several F2 or recombinant inbred line populations according to peak SNP and phenotypes. Then, we could ascertain several crosses that can map the same QTL by linkage mapping and rapidly isolated gene from QTL by Ho-LAMap directly. (PDF 19 kb) [file 12915_2017_365_MOESM30_ESM.pdf]

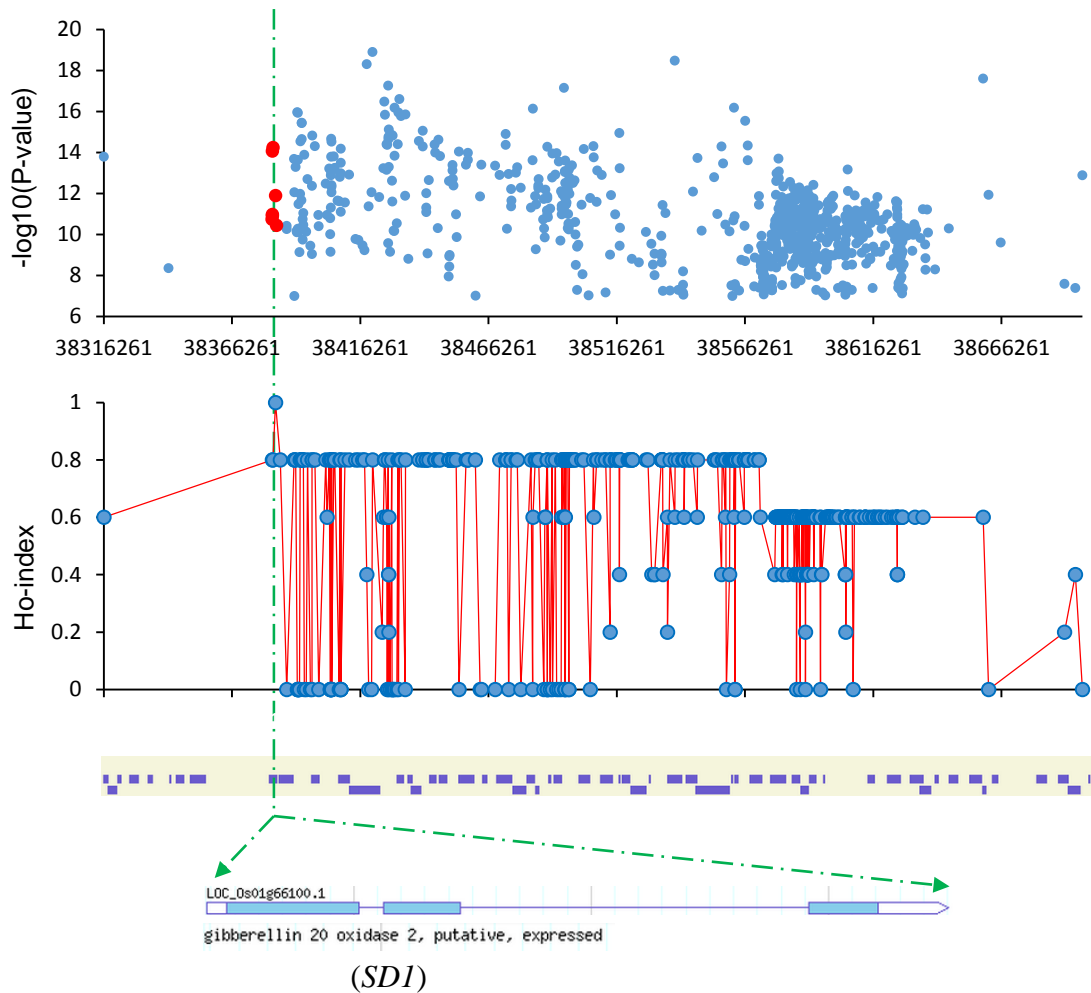

Supplement: Supplementary file 31 — Identification of the causal SNPs of QTL sd1 using Ho-LAMap. The top is Manhattan plot for candidate region association mapping for QTL region of sd1; the middle corresponds to Ho index plots for QTL region of sd1. The bottom corresponds to candidate gene (sd1), the green dashed lines label the region for significant signal. (PDF 175 kb) [file 12915_2017_365_MOESM31_ESM.pdf]
