# Supplementary material for: OsLG3 contributing to rice grain length and yield was mined by Ho-LAMap
Source: BMC Biol. 2017 Apr 6;15:28. doi: 10.1186/s12915-017-0365-7 (PMC5383996; doi:10.1186/s12915-017-0365-7)
Supplement: Supplementary file 33 — Notes S1. Natural population and linkage mapping (Additional file 2: Figure S2 and Additional file 11). Notes S2. Fine mapping of qGL3-3 (Additional file 10). Notes S3. Ho-LAMap – a potential method for validating association peak. Table S2. Summary of the taxa and source of 506 varieties of Oryza sativa L. Table S3. Environments used to evaluate association and linkage populations. Table S4. The heritability of grain traits in MCC1 Panel. Table S9. Summary of causal allele and type of variation about several grain size genes. Table S10. Grain phenotypes of six different parents. Table S11. Quantitative trait loci mapped in the four populations. Table S12. Simulation reveals the cross number need for several known genes (such as TGW6, GS3) about grain size when using Ho-LAMap. Table S13. OsLG3 polymorphisms associated with grain length in the MCC panel. Table S14. Comparison of polymorphisms between three large grain parents and three small grain parents. Table S16. Primers used for fine mapping and sequencing. Table S17. Primers used for DNA constructs and transcript analysis. (DOCX 73 kb) [file 12915_2017_365_MOESM33_ESM.docx]

**Additional file**

This file includes:

Notes S1-S3

Tables S2-S4, S9-S14 and S16-S17

(Tables S1, S5-S8 and S15 are provided in the separate excel files)

**Notes S1. Natural population and linkage mapping**

We measured grain size of the global rice Micro Core Collection [[1](#_ENREF_1)] (MCC1 panel) in five environments (Tables S1 and S2). Our MCC1 panel of 268 rice varieties (163 *indica*, 105 *japonica*) also displayed substantial and highly significant variation in grain size (Fig. S2 and Table S1), with high heritability, averaging 89% and varying from 82 to 97% (Table S4). GL ranged from 6.01 mm to 13.78 mm, GW ranged from 1.94 mm to 4.45 mm, and TGW ranged from 11.4 g to 58.8 g (Table S10). We observed moderate correlations between grain traits in the MCC panel (Fig. S11a). A significant population structure was identified and the *Indica* varietal group (*indica* and *aus* subpopulations) was significantly longer and more slender than the *Japonica* varietal group (consisting of the *temperate* *japonica*, *tropical* *japonica* and *aromatic* subpopulations) (p = 0.0001) (Fig. S4). One-way ANOVA demonstrated that subpopulations explained an average 72% of the phenotypic variation observed for grain size among the 232 accessions that carried a subpopulation classification. Despite the differences in mean grain size between subpopulations, considerable variation was also detected within each subpopulation.

Four hybrid combinations (BILs from NIP and SLG, CSSLs from YF and IRAT109, BC_1_F_2_ from CQ and SLG, and BC_1_F_2_ from NIP and HBK) were created. There were significant positive correlations between grain length and width or thickness in almost all populations (for example, 07DH014 (Fig S11b)), which was different from that of the MCC panel. Longer or wider grain also leads to heavier seed.

In summary, thirty-four QTLs for grain shape and weight were detected among the four combinations (Table S11). Grain shape in rice is under modestly complex genetic control, regulated by at least 29 QTLs, but represents one component of the more complex grain weight per se, with fewer genes controlling each component trait. Dissection of a complex trait into its components aids high-resolution genetic analysis.

**Notes S2. Fine mapping of *qGL3-3***

For developing NILs for the *qGL3-3* locus, a cross involving *japonica* cultivars, SLG-1 (SLG, donor parent) and Nipponbare (NIP, recurrent parent) was selected. SLG, a very large grain *japonica* variety (1,000-grain weight, 58.8 g), which is an improved temperate *japonica* and infiltrated chromosome segments from *tropical* *japonica*; whereas NIP is a small grain *temperate* *japonica* variety (1,000-grain weight, 23.3 g; Fig. S7). Based on the primary mapping results, plants homozygous for the *qGL3-3* locus were selected from more than 1,000 BC_4_F_3_ plants by SSR markers. We surveyed them using more than 100 SSR markers evenly distributed on all 12 rice chromosomes and obtained one plant (BC_4_F_3_-68-59) homozygous for the target region and containing the least genetic background from SLG (about 9.5% of the homozygous markers were from SLG). A total of 2,000 BC_5_F_2_ plants derived from BC_4_F_3_-68-59 were panted in the rice-growing season of 2013 at the China Agricultural University experiment farm. Using these data, *qGL3-3* was mapped into a 280 kb region between RM14484 and RM1278 (Fig. S10).

**Notes S3.** **Ho-LAMap - a potential method for validating association peak**

Here we propose a general approach for detecting genes underlying complex grain traits in rice. We explain the principle of this general approach for gene discovery from CrAM studies based on linkage mapping in rice by limiting the major problem caused by false positives (See Fig. S12).

In our experiment design, ideal populations for linkage mapping should be NAM population because of its many advantages in rice [[2-4](#_ENREF_2)] and it is most important that they adequately identify most of both major effect and minor effect QTLs. Our team has completed construction of about 100 RIL populations based on our MCC panel. It also indicated that our design might be an effective method to dissect the genetic architecture of grain traits and it has not only identified almost all known major effect QTLs, but also many minor effect QTLs for grain length. We have achieved some success, regarding *OsLG3*, *OsLG3b*, *GS*3 and *sd1* (Fig. 1d, Fig. S15, Fig. 2a and Fig. S31).

We also note some limitations of this Ho-LAMap. First, skimpy number of founder parents that could detect the target QTL might limited resolution to gene discovery. Second, there can be no assurance that founders include all type of alleles for all target traits. Third, here we didn’t find the insertion-deletions and structural variation, such as copy number variation (CNV) and presence-absence variation (PAV) among rice varieties, which may have functional importance and thus contribute to the variation that is not captured by SNPs.

The resolution of our method can reach the level of a single gene, primarily because the use of multiple well-chosen crosses should artificially weaken the degree of LD. In fact, on the way to cloning of *OsLG3* in rice, *qGL3-3* was narrowed down to one single gene just based on four crosses. Obtaining of unimaginable good result, one of the most important factors was that crosses for QTL scanning mainly were *japonica*×*japonica* populations and varieties within a single subspecies have more similar genetic backgrounds that reduce the confounding of excessive background variation that would be obtained using two subspecies. Thus we need to balance the trade-offs of choosing founder parents that contained similar genetic backgrounds and had an abundance of natural allelic variation.

**Supplementary References**

1. Zhang H, Zhang D, Wang M, Sun J, Qi Y, Li J, Wei X, Han L, Qiu Z, Tang S: **A core collection and mini core collection of Oryza sativa L. in China.** *Theoretical and Applied Genetics* 2011, **122:**49-61.

2. Buckler ES, Holland JB, Bradbury PJ, Acharya CB, Brown PJ, Browne C, Ersoz E, Flint-Garcia S, Garcia A, Glaubitz JC, et al: **The genetic architecture of maize flowering time.** *Science* 2009, **325:**714-718.

3. Kump KL, Bradbury PJ, Wisser RJ, Buckler ES, Belcher AR, Oropeza-Rosas MA, Zwonitzer JC, Kresovich S, McMullen MD, Ware D, et al: **Genome-wide association study of quantitative resistance to southern leaf blight in the maize nested association mapping population.** *Nat Genet* 2011, **43:**163-168.

4. Tian F, Bradbury PJ, Brown PJ, Hung H, Sun Q, Flint-Garcia S, Rocheford TR, McMullen MD, Holland JB, Buckler ES: **Genome-wide association study of leaf architecture in the maize nested association mapping population.** *Nat Genet* 2011, **43:**159-162.

**Tables**

**Table S2** Summary of the taxa and source of 506 varieties of *Oryza sativa L.*

| Sources | Types | *Indica* | *Japonica* | *Adm* | *Aus* | *Aromatic* | Total |
| --- | --- | --- | --- | --- | --- | --- | --- |
| China | Landrace | 64 | 45 | 6 | 1 | 0 | 116 |
|  | Improved | 138 | 60 | 6 | 1 | 0 | 205 |
| Foreign | Landrace | 27 | 28 | 2 | 1 | 6 | 64 |
|  | Improved | 77 | 32 | 6 | 2 | 4 | 121 |
| Total |  | 306 | 165 | 20 | 5 | 10 | 506 |

Note: Adm, admixture subpopulation; Landrace, landrace variety; Improved, improved variety.

**Table S3.** Environments used to evaluate association and linkage populations.

| Population |  | Environment | Longitude and latitude | Mean day lengths (h) |
| --- | --- | --- | --- | --- |
| Association panel | MCC1 | Sanya, Hainan, 2010 | E109.3 , N18.1 | 10.3 |
|  | MCC1 | Sanya, Hainan, 2012 | E109.3 , N18.1 | 10.3 |
|  | MCC1 | Changsha, Hunan, 2013 | E112.6 , N28.1 | 13.3 |
|  | MCC1 | Sanya, Hainan, 2013 | E109.3 , N18.1 | 10.3 |
|  | MCC1+MCC2 | Sanya, Hainan, 2014 | E109.3 , N18.1 | 10.3 |
| Linkage populations |  | Beijing,2012 | E116.2 , N39.5 | 14.7 |
|  |  | Sanya, Hainan, 2012 | E109.3 , N18.1 | 10.3 |
|  |  | Beijing,2013 | E116.2 , N39.5 | 14.7 |

**Table S4.** The heritability of grain traits in MCC1 Panel.

| Traits | Broad-sense heritability (%) |
| --- | --- |
| Grain length | 88 |
| Grain width | 90 |
| Grain thickness | 82 |
| Grain weight | 97 |

**Table S9.** Summary of causal allele and type of variation about several grain size genes.

| Gene | Causal variations | Change in protein or expression | Effect | Type of variation | Reference |
| --- | --- | --- | --- | --- | --- |
| *GS2* | TC487-488AA | Ser163Lys, the OsmiR396 target site | Enhances grain size and grain yield in rice | Rare allele | Hu et al.2015, Molecular plant |
| *GL3.1* | 1092C-A | Asp364Glu | Causes extra-large grain and a significant yield increase in rice | Rare allele | Qi et al.2012, Cell Research |
| *TGW6* | G/- | premature stop | Enhances rice grain length and weight | Common allele | Ishimaru et al.2012, Nature genetics |
| *GW8* | GAGCTGAGCT/---------- | lower expression | A loss-of-function mutation in Basmati rice is associated with the formation of a more slender grain | Common allele | Wang et al.2012, Nature genetics |
| *GS5* | Polymorphisms in the promoter | Higher expression | Higher expression of GS5 is correlated with larger grain size | Common allele | Li et al.2011, Nature genetics |

**Table S10.** Grain phenotypes of six different parents.

| Variety | GL | GW | GT | TGW | Variety group | Origin |
| --- | --- | --- | --- | --- | --- | --- |
| SLG-1 | 13.78 | 4.27 | 3.02 | 58.8 | *Temperate japonica* | Japan |
| Haobuka | 10.43 | 4.45 | 2.61 | 43.5 | *Temperate japonica* | Yunnan, China |
| IRAT109 | 9.01 | 3.91 | 2.37 | 34.2 | *Tropical japonica* | Africa |
| Nipponbare | 7.27 | 3.03 | 2.09 | 23.3 | *Temperate japonica* | Japan |
| Chuanqi | 6.01 | 2.48 | 1.68 | 11.4 | *Indica* | China |
| Yuefu | 6.83 | 3.20 | 2.11 | 22.9 | *Temperate japonica* | Beijing, China |

Note: GL, grain length; GW, grain width; GT, grain thickness; TGW, thousand-grain weight.

**Table S11.** Quantitative trait loci mapped in the four populations.

Note: Chr, Chromosome; QTL, Quantitative trait loci; GL, Grain length; GW, Grain width; GT, Grain thickness; TGW, Thousand grain weight. NIP, Nipponbare; SLG, SLG-1; CQ, Chuanqi; YF,Yuefu; IR109, IRAT109; HBK, Haobuka.

**Table S12.** Simulation reveals the cross number need for several known genes (such as *TGW6, GS3*) about grain size when using Ho-LAMap.

| Gene | Detected power | | | |
| --- | --- | --- | --- | --- |
|  | 60% | 80% | 90% | 100% |
| TGW6 | 3 | 4 | 5 | 10 |
| GW8 | 4 | 5 | 6 | 7 |
| GS3 | 6 | 8 | 10 | 18 |

**Table S13.** *OsLG3* polymorphisms associated with grain length in the MCC panel.

| Polymorphic site | R^2^(%)^a^ | P ^b^ | Ho-index ^c^ | Site |
| --- | --- | --- | --- | --- |
| InDel 1 | 11.2 | 3.59E-08 | 1 | -2358_-2354 |
| snp1 | 9.12 | 6.36E-10 | 1 | -2360 |
| snp2 | 10.18 | 6.25E-11 | 1 | -2365 |
| snp3 | 8.96 | 4.55E-09 | 1 | -2671 |
| snp4 | 9.00 | 3.67E-09 | 1 | -2915 |
| snp5 | 3.18E-04 | 0.780 | 0.5 | 926 |
| snp6 | 0.01 | 0.089 | 0 | 715 |
| snp7 | 0.01 | 0.080 | 0 | 392 |
| snp8 | 0.02 | 0.047 | 0 | 198 |

^a^ R^2^ values from ANOVA analysis of grain length showing % phenotypic variation explained.

^b^ p-value from candidate region association analysis for *qGL3-3*.

^c^ Ho (observed heterozygousity per locus) index as the ratio between the number of heterozygous cross corresponding to each SNP loci and the total number of crosses which have detected targeted QTL.

**Table S14.** Comparison of polymorphisms between three large grain parents and three small grain parents.

| Parents | Promoter region | | | | | Code region |
| --- | --- | --- | --- | --- | --- | --- |
|  | InDel 1 | snp1 | snp2 | snp3 | snp4 | snp5 |
| SLG | TTGTG | T | C | C | G | G |
| IRAT109 | TTGTG | T | C | C | G | A |
| HBK | TTGTG | T | C | C | G | A |
| YF | - | A | A | A | T | A |
| NIP | - | A | A | A | T | A |
| CQ | - | A | A | A | T | A |

Note: NIP, Nipponbare; SLG, SLG-1; CQ, Chuanqi; YF, Yuefu; HBK, Haobuka.

**Table S16.** Primers used for fine mapping and sequencing.

| Name | Forward primer (5' - 3') | Reverse primer (5' - 3') | Purpose |
| --- | --- | --- | --- |
| RM3392 | GTCCAATGATTCGTTCCCAC | CTTCACCGTTCACCAATTCC | Mapping |
| RM14484 | CTACTTCTCCTGTCCAAATCATCG | CGTCTCATTTCTTGATCTCTACCG | Mapping |
| RM1278 | CCATAGCAATTTAGCCATAT | TCTAATTCTCCCCAACACTA | Mapping |
| RM545 | CCTTCCCTGAAAGTATTCGTTCTCC | GAGAACGTCTTCATTGGATGTTCC | Mapping |
| M2 | GAAACGGGGGAGTAGAA | CAGCCGTGAATGGTGAC | Mapping |
| M7 | AAACGAGACCCACGACATA | TTTATTTTAGAGCCTTTGTTTC | Mapping |
| OsLG3-p1 | ACTGCTTGCCTCCCGCTAT | GGTGTTCTAAAAGTGTGAT | Sequencing |
| OsLG3-p2 | CACTTGGGTTGAATCAGT | TTCCTCTCACAGTATCACCCTT | Sequencing |
| OsLG3-p3 | AGAGGAGAAAGGGTGAT | AAAGTGCGGGTGGACAA | Sequencing |
| OsLG3-p4 | AAGTTGACACCTTGATGCT | CGAATGCCTTTGTTCTTGT | Sequencing |
| OsLG3 | GATTCAAACAGAGGCGAGAC | ATCATTAACAACCACGACCAC | Sequencing |
| OsLG3-p6 | CTCGGTTACAAGAACAAAGGC | GACGAAGCCGTTGGTGAA | Sequencing |
| GS3-FNP | ATTGGCTTGATTTCCTGTGC | TGCTCTTACGGGAGGACAT | Sequencing |
| GW8-FNP | CAAGGGAAGCAGAAACCCAGCAA | GCAGCCGATGACGACGATACCG | Sequencing |
| TGW6-FNP | GAGAATGTTCAAGACCA | AACTCGCATCAATCCCA | Sequencing |
| GW2-FNP | CCTTGGCAGTTGGCAATCTA | GGTATGACGGCACTGAAACA | Sequencing |

**Table S17.** Primers used for DNA constructs and transcripts analysis.

| Name | Forward primer (5' - 3') | Reverse primer (5' - 3') | Purpose |
| --- | --- | --- | --- |
| OsLG3-OE | CGGGGTACCAAAGGCATTCGCAACACACA | CCTTAATTAACCAAAATACATTACGACTGGAC | overexpression construct |
| OsLG3-RNAi | GGGGTACCACTAGTAAAGGCATTCGCAACACACACT | GGGGATCCGAGCTCCCCCGCTTCTTCTTGCTCG | RNAi construct |
| OsLG3-GFP | CCTTAATTAACAATGTGTGGAGGCGCCATC | CGGGGTACCCAGAAAAGGGCGCCGTCGATT | 35S:OsLG3:GFP construct |
| OsLG3-GUS | CGGGGTACCAGAGCACAATAGGAGTAGGGTA | TTGGCGCGCCGGGTCTCCCACTTCTCTTCG | OsLG3:GUS construct |
| OsLG3-A1 | CGCGGATCCGTATGTGTGGAGGCGCCATC | AACTGCAGATCAGAAAAGGGCGCCGTC | transactivation assay |
| OsLG3-A2 | CGCGGATCCGTATGTGTGGAGGCGCCATC | AACTGCAGGGGGAAGTTGACCTTGGCCTTG | transactivation assay |
| OsLG3-A3 | CGCGGATCCGTATGTGTGGAGGCGCCATC | AACTGCAGGCTTCTTCTTGCTCGCCGCCCT | transactivation assay |
| OsLG3-A4 | CGCGGATCCGTCGGGGGCGGCACTTCCGAGGCA | AACTGCAGATCAGAAAAGGGCGCCGTC | transactivation assay |
| OsLG3-A5 | CGCGGATCCGTCCACGCCGCGGCAACACGAGA | AACTGCAGATCAGAAAAGGGCGCCGTC | transactivation assay |
|  |  |  |  |
| OsLG3-RT | ATGGCTTGCTTGATTACCGAA | AGACCCCGTAAAAGTAGCCCA | qPCR |
| GW2-RT | CAgCAgCgCATTCCCAgTTTTC | gTggTCAgCCgAgCACTCTC | qPCR |
| GS3-RT | CATCggAgAAgCgAAgTCAT | TTgAggTTgAAggAggAggA | qPCR |
| qPPKL1-RT | TAACAACATGACGCCTCCACCTG | GTTTCTGCAGCCACTACAACAGC | qPCR |
| TGW6-RT | TTGAACTTGCAAAAGGCAGA | CGGTTCCCCTAATGCAGAT | qPCR |
| GW6a-RT | CGTGTATAAATGCGCCACAC | GGCCGATCTCACCAGCTAC | qPCR |
| GW8-RT | AggAgTTTgATgAggCCAAg | gCgTgTAgTATgggCTCTCC | qPCR |
